# Experience of the COVID-19 Pandemic in Rural Nigeria: A Scoping Review of the Literature Contextualized With Local Knowledge Using Fuzzy Cognitive Mapping

**DOI:** 10.1177/2752535X251384511

**Published:** 2025-10-06

**Authors:** Mona Z. Ghadirian, Iván Sarmiento, Natalia Reinoso Chávez, Neil Andersson, Anne Cockcroft

**Affiliations:** 1 CIET-PRAM, Department of Family Medicine, 5620 McGill University, Montréal, Canada; 2 Grupo de Estudios en Sistemas Tradicionales de Salud, Escuela de Medicina y Ciencias de la Salud, Universidad del Rosario, Bogotá, Colombia; 3 Centro de Investigación de Enfermedades Tropicales, Universidad Autónoma de Guerrero, Acapulco, México

**Keywords:** COVID-19 pandemic, participatory models, participatory research, socioeconomic recovery, equity, weight of evidence

## Abstract

**Aim:**

Collate and summarise published evidence of the non-clinical effects of the COVID-19 pandemic in rural Nigeria and compare the findings with community stakeholder experiences.

**Methods:**

We searched PubMed, Scopus, and Cumulative Index to Nursing and Allied Health Literature (CINAHL) for peer-reviewed papers published up to January 2024. Included studies used quantitative, qualitative, or mixed methods to examine the influence of the COVID-19 pandemic on the lives of rural Nigerians. Two reviewers conducted title, abstract, and full-text screening independently. We used narrative descriptions and fuzzy cognitive maps to summarise the findings of the review and compared the maps with those previously created by stakeholders in rural communities in Bauchi State, rural Nigeria.

**Results:**

Poverty, hunger and lack of food, and stress and mental health problems were leading themes in both the literature and stakeholder maps. Stakeholder maps highlighted job loss and household conflicts. These topics were rarely explored in the literature, which emphasized reduced health services.

**Conclusion:**

This review and stakeholder perspectives confirm the importance of non-clinical impacts of the COVID-19 pandemic in rural Nigeria. Some issues highlighted by local community stakeholders were absent in the literature. Contextualizing published research with local experience provides specific insights to inform recovery policies.

## Introduction

The impact of the COVID-19 pandemic on sub-Saharan Africa has been far-reaching and complex.^1^ Focusing solely on health impacts of the infection provides an incomplete picture. Secondary effects of the pandemic bring long-term challenges for individuals and communities. The United Nations emphasized the need to study the indirect impacts of the pandemic to inform comprehensive and equitable recovery strategies.^2^ The pandemic’s disruptions to economic activities, education, food systems, and healthcare services exacerbated pre-existing vulnerabilities, particularly in low-resource settings.^3,4^ Stringent lockdown and social distancing measures contributed to market closures, job losses, and weakened local economies.^1,3^ These changes led to steep increases in the prices of food and goods, increasing poverty and food insecurity.^5^ Pre-existing structural and socio-economic inequalities exacerbated these challenges, disproportionately affecting marginalized populations and further widening inequalities.^6^ This review examines the pandemic’s secondary effects on rural Nigerian populations to gain insights that may inform ongoing COVID-19 recovery strategies in Nigeria and other low-income countries.

Nigeria is Africa’s largest economy, yet nearly 40% of its population live in poverty; national statistics mask marked regional health and socioeconomic inequalities.^7^ Access to resources and health outcomes are worse in rural settings. Rural Nigeria has poor infrastructure, fragile healthcare systems, and relies on informal economies.^7,8^

There were relatively few deaths from COVID-19 in Nigeria during the pandemic, even allowing for serious under-reporting of cases: in January 2023, Nigeria (with a population of over 218 million) reported 266,207 COVID-19 cumulative cases and 3155 deaths.^9^ But pre-existing inequalities were exacerbated by market closures, job losses, and mobility restrictions, which weakened local economies and restricted access to basic needs.^1,3,10,11^ The shift in healthcare resources toward COVID responses disrupted services such as antenatal care and immunizations.^12^ School closures reduced access of millions of children and adolescents to education, extra-curricular activities, and social interaction.^13,14^ Lockdown and social distancing disrupted social networks, contributing to stress and mental health problems.^15^ Many small studies have reported on impacts of the pandemic on specific, usually researcher-defined, outcomes in rural Nigeria.

Our scoping review collates the published evidence about these impacts. It is part of a larger project examining the impact of the COVID-19 pandemic in rural Bauchi State, Nigeria, and supporting community co-design of recovery strategies. We used fuzzy cognitive mapping (FCM) to compare the evidence from the scoping review with community stakeholder perspectives about the factors that worsened their experience of the COVID-19 pandemic. FCM depicts perceived causal relationships.^16^ It provides a common format for juxtaposition of different knowledge sources.^17^ The maps can depict relationships reported in the literature, associations identified through survey data, or causes perceived by stakeholders.^17^ Dion and colleagues developed the Weight of Evidence approach using fuzzy cognitive mapping to update and contextualise literature reviews with knowledge and perceptions of stakeholders.^18,19^ The approach was useful for contextualising the literature about the role of traditional midwives for maternal health in Indigenous communities in Mexico.^20^ We created cognitive maps of the factors related to the experience of the COVID-19 pandemic identified in the scoping review and compared them with community stakeholder maps of perceived causes of a bad experience of the pandemic in Bauchi State, northern Nigeria.^21^

## Methods

### Overview

We conducted a scoping review of the literature, reporting according to the Preferred Reporting Items for Systematic Reviews and Meta-Analyses for Scoping Reviews (PRISMA-ScR) (see Supplemental Material).^22^ Following the Joanna Briggs Institute guidelines, we framed our research question using the People, Concept and Context model: What contributed to a negative experience of the COVID-19 pandemic for rural Nigerians (Table)?^23^ We extracted data from the selected articles in a way that allowed us to create fuzzy cognitive maps to summarize the results and compare with the community stakeholder maps from Bauchi State.

**Table 1.** Participants, Concept, and Context.

Criteria	Description
Participants	Anyone residing in rural Nigeria, irrespective of characteristics such as age, sex, gender, religious or ethnic background.
Concept	Any impact of the COVID-19 pandemic on health and wellbeing, other than morbidity and mortality due to coronavirus infection.
Context	Rural Nigeria

### Search Strategy

We searched for peer-reviewed articles in PubMed, Scopus, Cumulative Index to Nursing and Allied Health Literature (CINAHL) published before January 2024. The search matrix included a combination of keywords to represent the rural Nigerian population and search terms related to the COVID-19 pandemic. Supplemental Material presents the search matrices we used.

### Eligibility Criteria

We included studies about the impact of COVID-19 in rural Nigerian populations. We excluded papers reporting exclusively on morbidity or mortality due to coronavirus infection. The review included articles published in English peer-reviewed journals at any time up to January 2024. Eligible articles presented empirical evidence from qualitative, quantitative, or mixed methods studies. We considered multinational studies but excluded articles that did not present disaggregated data from rural Nigerian settings. We excluded reviews, editorial pieces, and grey literature, such as reports, conference proceedings, or other non-peer-reviewed documents.

### Study Selection

Two reviewers (MZG and NRC) independently screened titles and abstracts. They resolved disagreements through discussion. They screened the full text of potentially eligible articles according to the agreed eligibility criteria, recording why they excluded articles and resolving disagreements through discussion, involving a third reviewer (IS) if necessary.

### Data Extraction

MZG and NRC independently extracted data from included articles, involving a third reviewer (IS) if necessary to resolve disagreements. They created a data extraction template in Airtable (airtable.com), piloted it with five randomly chosen studies, and made modifications.

The template recorded study characteristics (authors, year of data collection, study design, data collection methods, research setting) and participant demographics (gender, age, occupation). It also recorded the reported impacts of the COVID-19 pandemic in a form to support creation of fuzzy cognitive maps from the review. The guidelines for identifying and justifying relationships extracted from the literature are shown in Supplemental Material. We specifically trained reviewers for the task. Two reviewers undertook the task separately and resolved conflicts by discussion with a third reviewer. For each identified relationship, the reviewers recorded the supporting data from the paper. For quantitative studies, they included all relationships significant at the 5% level in univariate analysis or in multivariate analysis if available. For qualitative studies, they recorded quotes describing the relationships. They did not include relationships based on assumptions or hypothesized explanations reported in the papers. They included second order relationships when these were reported. For example, a paper might report that lockdown caused market closures, which caused food insecurity.

From the extracted data on relationships, the reviewers created a list of reported relationships (an edge list). Edge lists are tabular formats of the elements of fuzzy cognitive maps. The first column contains the factors originating relationships (causes), the second column contains the landing factors (outcomes), and the third column indicates the relationship weights and signs.^20^ Positive signs indicate that an increment of the cause leads to an increment in the outcome, while negative signs indicate that an increment of the cause leads to a decrease in the outcome. For the literature maps, any reported relationship received a weight of (meaning it was present in the study). For example, a qualitative study might report that market closure (cause) resulted in food insecurity (outcome). The relationship sign is positive because more markets closure leads to more food insecurity.

### Data Analysis and Mapping

The research team standardized the names of the factors reported across the studies, ensuring factors with effectively the same meaning had the same name. On the standardized maps, we applied fuzzy transitive closure (available in CIETmap 2.2).^24^ This mathematical model translates the map of individual associations into a knowledge network by identifying all the direct and indirect connections between factors and all possible ways a factor on the map can influence others.^24^ Transitive closure adjusts the map by down-weighting direct arrows it adds between nodes (factors) that previously only had indirect connections.

To weight each relationship in the literature map, we calculated its relative frequency across the studies in the review. Inspired by Harris’ original discourse analysis, the more articles that identify the same relationship, the more likely the causal meaning of the relationship within the “universe” of the literature.^25^ This weighting process is operator-independent: it does not rely on researcher interpretation to assign weights to relationships.^26^

To simplify interpretation, four of the authors (MZG, IS, NRC, and AC) condensed the aggregate literature map into a category map, grouping factors with similar implications for action. We categorized using a digital version of the physical grouping of factor labels on a large whiteboard used for community stakeholder categorization in Nigeria. Considering factors one by one, the authors placed related factors together while constantly reviewing the emerging categories. We first used categories developed from community stakeholder FCM in Bauchi State.^21^ We created additional categories for factors that did not fit into any of these categories.

We used social network analysis to compute indegree and outdegree centrality for each category in the map.^27^ Outdegree centrality is the sum of absolute values of the weights of outgoing arrows; higher values indicate more important causes in the network. Indegree centrality is the sum of absolute values of the weights of incoming arrows; higher values indicate important outcomes in the network.

### Comparing the Scoping Review with Local Knowledge Using FCM

We created pattern-matching tables contrasting outdegree and indegree centrality measures between the literature and stakeholder category maps. This highlighted the similarities and differences in the most important causes and outcomes between the two knowledge sources. We also compared the narrative content of categories in the literature and stakeholder maps.

## Results

### Included Articles and the Factors They Considered

Figure 1 presents the PRISMA flow diagram and outlines the selection process from 742 references to 33 studies for inclusion. Table 2 summarises the characteristics of the included articles. Seven described international studies that included disaggregated data for rural Nigeria.^31,37,38,53,55,56,60^ Most studies used quantitative methods, mainly cross-sectional designs (*n* = 23), seven used qualitative methods, and two applied mixed methods. The included studies collected data between 2020 and 2022; 14 studies looked at the pandemic’s early effects in 2020.

**Figure 1.**
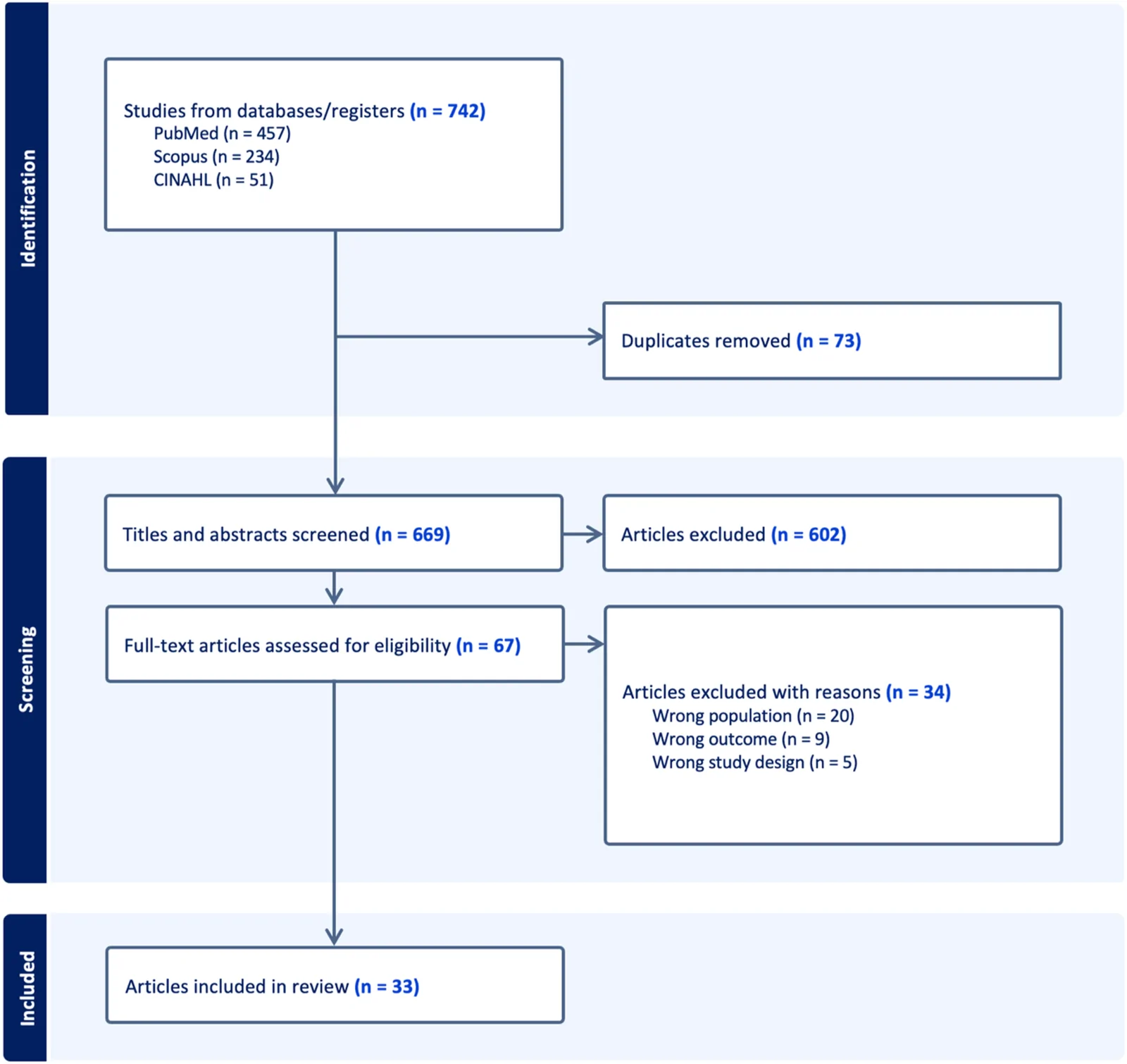
PRISMA flow diagram of scoping review on the impact of the COVID pandemic in rural Nigeria.

**Table 2.** Characteristics of Included Studies.

Ref.	Primary author, year of publication	Year(s) of data collection	Main topic of concern	Study design	Participants
^28^	Ajefu 2023	2020	Access to basic needs	Cross-sectional	Rural households
^29^	Akwaowo 2021	2020	COVID-19 perception and preventive practices	Cross-sectional	Adult community members
^30^	Amuzie 2023	2022	Unmet need for family planning during the postpartum period	Cross-sectional	Women of reproductive age (15-49 years) in a marital or sexual union
^31^	Assefa 2023	2021	COVID-19 perception and preventive practices, psychological distress, and disruption of health services	Cross-sectional	Adult community members
^32^	Ekoh 2020	2020	Social support of elders	Qualitative	Elders (60 years old and above)
^33^	Ekoh 2021A	2021	Experience of traditional leaders during pandemic	Qualitative	Traditional rulers
^34^	Ekoh 2021B	2020	Digital exclusion of elders	Qualitative	Elders (60 years old and above)
^35^	Ezeah 2020	2020	Knowledge/awareness of COVID-19 and use of interpersonal communication during the pandemic	Quasi-experimental	Adult community members
^36^	Iliyasu 2023	2022	Maternal health service utilization, predictors, and childbirth experiences	Mixed methods	Women of reproductive age (15-49 years) who gave birth during COVID-19 restrictions
^37^	Josephson 2021	2020	Socioeconomic impacts of the pandemic and mitigation policies	Longitudinal	Rural households
^38^	Maredia 2022	2020	Income and food consumption	Cross-sectional	Adult community members
^39^	Michael 2021	2020	Unmet need for family planning	Cross-sectional	Adult community members
^40^	Ndubuisi-Okoroezi 2022	Not declared	COVID-19 information-seeking patterns	Cross-sectional	Adult community members
^41^	Nnama-Okechukwu 2020	2020	COVID-19 knowledge and compliance with preventive measures	Qualitative	Adult community members
^42^	Ojeka-John 2023	Not declared	Government communications about COVID-19 risk to non-literate populations	Cross-sectional	Non-literate adult community members (aged5 years and above)
^43^	Ojo 2023	2021-2022	COVID-19 vaccine uptake	Cross-sectional	Rural households
^44^	Olanrewaju 2021	2021	Digital limitations for e-learning in rural secondary schools	Mixed methods	Secondary school students (aged4 years and above)
^45^	Omoronyia 2020	2020	COVID-19 knowledge and preventive practices	Cross-sectional	Community health workers
^46^	Onukwuli 2023	2021	Adolescent internet addiction	Cross-sectional	Secondary school students
^47^	Orjiakor 2023	Prepandemic, 2020, 2021	Household food insecurity	Longitudinal	Rural households
^48^	Osarenkhoe 2023	2022	COVID-19 vaccine awareness, attitude, and uptake	Cross-sectional	Adult community members
^49^	Owhonda 2022	2020	COVID-19 awareness, perception, and preventive practices	Cross-sectional	Adult community members
^50^	Oyekale 2022	2020-2021	Youth’s career aspirations and willingness to take COVID-19 vaccine	Cross-sectional	Youth (15–25 years old)
^51^	Oyekale 2023	2021-2022	Health insurance and willingness to take COVID-10 vaccine	Cross-sectional	Unvaccinated respondents in the021/2022 National Longitudinal Phone Survey (NLPS)
^52^	Rasaq 2023	2021	Unvaccinated people’s perception of COVID-19 infection and vaccine, and vaccine willingness	Cross-sectional	Heads of households
^53^	Rudin-Rush 2022	2018-1019, 2020, 2021	Food security over time	Longitudinal	Rural households
^54^	Sato 2022	2017, 2020	Trust in government and COVID-19 hesitancy	Cross-sectional	Rural households
^55^	Wang 2023	2021	Adolescent mental health and education	Cross-sectional	Adolescents (10-20 years old)
^56^	Wang 2022	2021	Adolescent COVID-19 vaccine hesitancy	Cross-sectional	Adolescents (10-19 years old)
^57^	Yahemba 2023	2020-2021	Community health worker challenges (workload, stigma, community relationships)	Qualitative	Community health workers
^58^	Ekoh 2022	2020	Pandemic’s influence on the social lives of elders	Qualitative	Elders (60 years old and above)
^59^	Reghagwa 2021	Not declared	Elders’ access to health information	Cross-sectional	Elders (65 years old and above)
^60^	Wang 2021	2020	Adolescents’ COVID-19 knowledge, perception, and preventive practices	Cross-sectional	Adolescents (10-19 years old)

A common focus of the included articles was COVID-19 knowledge, perceptions, and preventive practices. Seven articles focused on individual experience of understanding and complying with public health measures and the influence of these measures on personal, economic, and community life.^29,31,35,41,45,49,60^ Five articles addressed vaccine hesitancy, including awareness about the vaccine, willingness to receive the vaccine, and trust in government communications.^43,48,50,51,52,54,56^ Four studies focussed on socioeconomic impacts of the pandemic, including food insecurity, income loss, and restricted access to basic needs.^28,38,47,53^ Three studies examined the challenges elders and adolescents faced in using digital technology to facilitate social contact, access information, and participate in e-learning platforms during the pandemic.^34,44^ Three studies focused on maternal and reproductive health, examining unmet family planning needs and barriers to accessing maternal health services.^30,36,39^ Other topics included adolescent mental health, community health worker challenges, and the social impacts on older adults.

### Factors and Categories in the Literature Maps

The 33 articles each included an average of nine relationships (range 1–50), totalling 388 relationships across all maps after fuzzy transitive closure (see Supplemental file 4). The studies identified 227 standardized factors that we grouped into 29 categories (see Supplemental file 5). Table 3 summarizes the content of each category.

**Table 3.** Brief Description of Categories of Factors From the Literature.

Category	Description
Fear and misconception about COVID (C01)	This category included community awareness about COVID-19 and its preventive measures (including COVID-19 vaccine), the forms of COVID information/communication, distrust in government, and fear and stigma around contracting or spreading the virus. Factors included misunderstanding and disbelief about the existence of COVID-19, knowledge about preventive measures, and fear and distrust in vaccinations and its side effects. This category included factors around COVID-19 communications such as information source credibility, distrust in government, health organizations and medical doctors, use of awareness campaigns, relatable COVID communications, conflictive communications about the pandemic, communication being provided in native tongue, and inadequate support from government to boost access to health information. This category included perceived susceptibility to COVID19 infection, fear and stigma that others would think you are COVID positive, as well as a fear contract and spread the virus friends and family.
Lockdown and control measures (C02)	Most articles referred to government restrictions, policies, and strategies to prevent the spread of COVID-19. Articles referred to more stringent and prolonged COVID-19 restrictions and control measures such as the lockdown, movement restrictions, closure of state borders, lack of transport as playing an important role on limiting movement and daily activities.
Poverty, hunger and lack of food (C03)	This category encompasses the widespread economic and financial challenges during the pandemic. It included factors that highlighted elders’ risk of poverty. Elders were dependent on intergenerational support and often lacked pensions, while their children also struggled financially. This category also included reduced affordability of preventative tools, financial strain due to injury or illness, lack of resources and infrastructures like electricity and transportation. It also included factors like household income levels, health insurance. This category also referred to the issue of food insecurity. It included the challenges to afford and store food safely, shortages in food supply, and access to staple foods. This category also examined food consumption patterns, such as the consumption of animal-sourced foods among adolescents, or households skipping meals due to lack of food.
Access to communication modalities (C04)	The category includes access to digital technologies like smartphones, limited internet access, and seeking information about COVID-19, and knowledge acquired through migrant networks. This also includes factors specific to the elder population, such as their information seeking through mass media, family members, friends, or physicians, their access to radio and non-digital technologies, and their use of digital technology despite challenges like low digital literacy, and lack of interest in new technology.
Reduced health services (C05)	This category focused on challenges to provide and access health services and disruptions to routine care during the pandemic. Health services and medical training focused on COVID-19 in health facilities at the expense of other health conditions. Community health workers were unable to provide routine care. There were delays in campaigns to control Neglected Tropical diseases and other education and immunization campaigns. This category included reduced utilization and access of health services such as maternal and antenatal care. It also included the unmet medical needs such as compromised immunization in the community, unmet needs in family planning for women in extended postpartum period, and maternal care.
Reduced businesses and jobs (C06)	Reduced businesses and jobs referred to a range of disruptions to economic activity such as closures of non-farm businesses, shops, and markets, farming disruption, and changes in market diversity. Additionally, rural markets were more vulnerable to disruptions in urban areas when they were highly dependent and connected to them (highly interdependent rural-urban linkage). This category included loss in income source, and decreased income and remittances.
Difficulties for community health workers (C07)	Community health workers experienced a range of challenges during the pandemic, such as increased workload and exposure to COVID-19 cases, personal loss and bereavement, and added costs for health workers. This category also includes health workers challenges with distance from their families, undone home chores, lack of time, and lack of rest. Community health workers and volunteers spoke of the influence of the pandemic on their relationships with the community and the availability of resources like transport, personal protective equipment, and lab equipment.
Traditional rulers’ protective role (C08)	This category examines factors related to traditional rulers’ role in the community. It includes factors such as traditional rulers’ involvement in COVID-19 prevention measures, encouraging protective measures during ceremonies, or distributing relief materials from the government. It also includes traditional rulers’ perceived ability to ensure community safety and to raise awareness about the coronavirus, serving as role models in facing COVID-19.
Rural communities/population (C09)	Some studies examined how rural Nigerian communities were affected by the pandemic by comparing them to their urban counterparts. This category includes studies that explicitly analyzed differences between rural and urban Nigerian communities.
Reduced education (C10)	Rural schools were closed in the pandemic and their was limited access to digital learning platforms. Students fell behind in their studies or received no formal education during the pandemic. This category also includes adolescents’ struggles with digital literacy, awareness of digital learning interventions, preference for face-to-face learning, and perceived lack of teacher support for e-learning. Mitigating factors were informal learning channels like radio or parents teaching their adolescents alternative skills.
Reduced social contact and activities (C11)	This category includes reduced opportunities for social engagement and out-of-home relationships. It includes inability to attend social gatherings (worship, cultural events) and reduced social contact with friends, family, and community members. Some studies focused on older people, especially those living alone, who had reduced social contact with family and children, and reduced intangible assistance. The category also includes reduced adolescent communication with family and friends outside the home, particularly during school closures.
Price hike (C12)	Increased prices of basic goods, such as food and farming inputs.
Enforced social distance (C13)	This category refers to government regulations on social distancing and the ban on social gatherings.
Level of schooling (C14)	This category includes the positive effect of increased education among both men and women, and the negative influence of illiteracy among rural residents.
Preventive behaviours (masks and handwashing) (C15)	This category includes community efforts to adopt preventive practices during the pandemic. It includes wearing masks and hand washing, as well as access to cleaning supplies and soap.
Older age (C16)	Articles examined the influence of older age, including men over5 and retirees.
Adolescents (C17)	Articles examined the influence of the pandemic on adolescents’ behavioural patterns, including adolescent students.
Migration within states (C18)	Some articles considered the experience of migrants. This category highlights rural woman migrants and the risks associated with moving across and within state boarders.
Reduced government support (C19)	Reduced government support includes mainly limited distribution of palliatives in rural settings. Some studies focused on elders’ limited access to palliatives and lack of consideration for their particular medical and nutritional needs.
Woman (C20)	Studies compared the different experience of women and men in the pandemic.
Lack of access to money (C21)	Difficulties accessing money included limited access to banking services and Automated Teller Machines (ATMs).
Children staying at home (C22)	During the pandemic children and youth stayed at home because schools were closed. This could be positive, with parents getting to know their children better or negative as their presence increased stresses at home
COVID-19 vaccination (C23)	This category refers to COVID-19 vaccine uptake and related challenges like the cost of the vaccine for the recipient, vaccine availability, and community willingness to take the vaccine.
Stress and mental health problems (C24)	Psychological distress and mental health problems affected various groups across the community. This category included feelings of loneliness, isolation, and exclusion, particularly among the elderly. It also included community health workers’ pride in their work, and well as their distress in school closures, and concern for their children’s education.
Reduced religious activities (C25)	Closure of religious institutions
Religious beliefs (C26)	This category refers to confidence in spiritual protection, so that individuals feel protected by their religious beliefs and practices.
Increased social vices (C27)	This category includes increases in antisocial behaviours such as adolescent negative media consumption and internet addiction. It also refers to increased crime.
Know someone with COVID-19 (C28)	Knowing someone who has contracted COVID-19 highlights the personal connection and awareness of the virus and its spread.
Change in gender roles (C29)	Gender roles were changing and being redefined during the pandemic, reflecting shifts in gender dynamics and evolving gender expectations. This category included women’s challenges in balancing their traditional roles as mothers and wives with work responsibilities.

Figure depicts the relationships in the literature category map between categories with the highest indegree centrality (outcomes) and highest outdegree centrality (causes). Online Supplemental File shows the adjacency matrix for the final literature category map showing all relationships and their weights, and the calculated indegree and outdegree centrality of each category.

**Figure 2.**
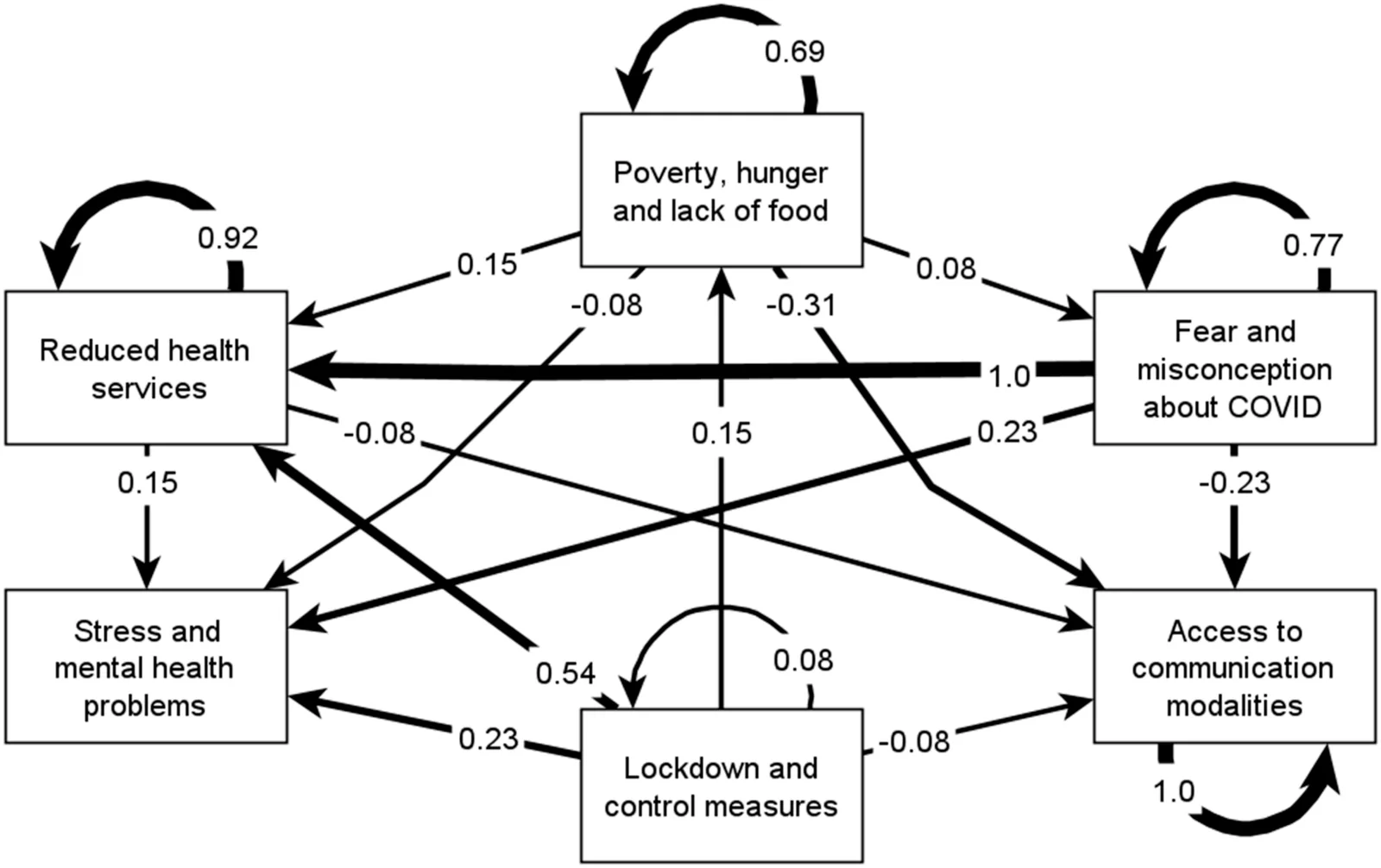
Relationships among the categories with higher indegree and outdegree centrality. This fuzzy cognitive map includes the five categories with the highest indegree and outdegree centrality, representing the main outcomes and causes in the literature map. The complete set of relationships is provided in Online Supplemental File 4. Line thickness is proportional to the cumulative influence of one category on another, with values of ±1 indicating the strongest influences.

### Main Outcomes in the Literature Map

Table 4 shows the main outcome categories in the literature (higher indegree centrality). Table 3 shows the contents of each category. The most prominent outcome was *reduced health services,* including challenges to providing and accessing health services and disruptions to routine care. The category included reduced utilization and access to health services such as maternal and antenatal care. Studies described unmet needs for immunization, family planning, and maternal health care.

**Table 4.** Rankings of the Five Categories With Highest Indegree Centrality in Literature and Stakeholder Maps.

	Indegree centrality ranking	
Categories	Literature map	Stakeholder map
Reduced health services	**1**	12
Poverty, hunger and lack of food	**2**	**2**
Stress and mental health problems	**3**	**4**
Fear and misconception about COVID	**4**	16
Access to communication modalities	**5**	not identified
Increased illness and death	not identified	**5**
Increase in household conflicts	not identified	**3**
Bad experience of COVID-19 pandemic	not identified	**1**

Categories with high indegree centrality are important outcomes within a network.

Categories ranked-5 for each source (literature or stakeholder) are shown in bold font. Categories below this ranking are shown in plain font.

“Bad experience of COVID-19 pandemic” was the central concern for which community stakeholders identified their perceived causes in their maps.

The second most prominent outcome (Table 4) was *poverty, hunger, and lack of food*. This category included increased poverty specifically among elderly people who depended on intergenerational support but whose children were also struggling financially. Food insecurity was prominent, with households skipping meals, unable to buy and store food, and facing staple food supply shortages.

*Stress and mental health problems* were ranked third among outcomes (Table 4). This category included feelings of loneliness, isolation, and exclusion, particularly among the elderly.

*Fear and misconceptions of COVID* (Table 4) ranked fourth as an outcome. This broad category included misconceptions about the existence of COVID-19 and fear and distrust around vaccination. Fear and misconceptions were reduced by effective awareness campaigns, and the protective role of traditional rulers. Fear and stigma concerned contracting COVID and spreading infection to friends and family.

*Access to communication modalities* (Table 4) was the fifth most prominent outcome. This outcome mitigated against adverse effects of other categories. Among older people, the category included actively seeking information, access to radio and non-digital technologies, and use of digital technology.

### Leading Causes in the Literature Map

Table 5 shows the main causes in the literature map, based on outdegree centrality. Table 3 shows the contents of each category. *Fear and misconception about COVID* was the first ranked cause of other categories in the map, especially *reduced health services.* Internal dynamics (relationships between factors within the category) reinforced the effect of this category. For example, lack of credibility of information sources reduced knowledge about COVID-19. Distrust in the government reduced willingness to be vaccinated.

**Table 5.** Rankings of the Five Categories With Highest Outdegree Centrality in Literature and Stakeholder Maps.

	Outdegree centrality ranking	
All categories	Literature map	Stakeholder map
Fear and misconception about COVID	**1**	6
Lockdown and control measures	**2**	**1**
Poverty, hunger, and lack of food	**3**	**3**
Access to communication modalities	**4**	not identified
Reduced health services	**5**	11
Reduced businesses and jobs	6	**2**
Increase in household conflicts	not identified	**4**
Reduced education	10	**5**

Categories with high outdegree centrality are important causes within a network.

Categories ranked-5 for each source (literature or stakeholder) are shown in bold font. Categories below this ranking are shown in plain font.

The close second ranked cause on the map was *lockdown and control measures.* The main associated outcomes were *reduced health services* and *lack of social contact and activities*. *Lockdown and control measures* contributed importantly to *stress and mental health problems.*

*Poverty, hunger and lack of food* ranked third as a cause (Table 5), linked with outcomes such as *stress and mental health problems*. The fourth-ranked cause was *access to communication.* This category reduced the outcome of *reduced education*, through improved internet connectivity.

*Reduced health services* was the fifth strongest causal category. The inability of community health workers to provide routine care compromised immunization efforts, and the focus on COVID-19 in health facilities significantly impacted healthcare services. Fear of harassment during childbirth, overcrowding in clinics, and unclean examination areas deterred individuals from seeking medical attention. Non-availability of health workers and maternal health services led to unmet needs for family planning.

*Reduced businesses and jobs* was the sixth strongest cause. This included disruption from closure of businesses, shops, and markets, and farming disruption. Loss of sources of income included loss of remittances from family members working away from home.

### Comparison of the Scoping Review Findings with Stakeholder Knowledge

Stakeholder maps created by Bauchi community members included 25 categories,^21^ 14 of which were also identified in the literature. Table 6 lists the unique and shared categories between the two knowledge sources. Community members in rural Bauchi State, Nigeria, were asked, “What made the COVID-19 pandemic a bad experience for people like you?” at the beginning of their FCM sessions.

**Table 6.** Comparison of Categories in Literature and Stakeholder maps: shared, Literature-exclusive, and Stakeholder-Exclusive Categories.

Categories	Shared	Only in literature map	Only in stakeholder map
Enforced social distance	1		
Fear and misconception about COVID-19	1		
Poverty, hunger and lack of food	1		
Increased social vices	1		
Lack of access to money	1		
Lockdown and control measures	1		
Price hike	1		
Reduced businesses and jobs	1		
Reduced education	1		
Reduced government support	1		
Reduced health services	1		
Reduced religious activities	1		
Reduced social contact and activities	1		
Stress and mental health problems	1		
Preventive behaviours (masks and handwashing)		1	
Access to communication modalities		1	
Adolescents		1	
Change in gender roles		1	
Children staying at home		1	
COVID-19 vaccination		1	
Difficulties for community health workers		1	
Know someone with COVID-19		1	
Level of schooling		1	
Migration within states		1	
Older age		1	
Religious beliefs		1	
Rural communities/population		1	
Traditional rulers’ protective role		1	
Woman		1	
Bad experience of COVID-19 pandemic			1
Animal/herders problems increased			1
Conflict between government and citizens			1
Faulty clinical isolation policies			1
Increase in early marriage			1
Increase in household conflicts			1
Increased illness/death (from non-COVID causes)			1
Lack of COVID-19 commodities			1
Penalties/punishment (related to control measures)			1
Problems with personal protective equipment			1
Reduced physical fitness			1

*Bad experience during the COVID-19 pandemic* was the most important outcome for stakeholders (Table 4) because this category was the central outcome for their mapping sessions. This outcome did not appear in the map from the literature; rather the articles examined outcomes and potential causes identified by researchers. Both sources of knowledge identified *poverty, hunger and lack of food* and *Stress and mental health problems* as prominent outcomes of the pandemic. However, the main outcome reported in the literature, *reduced health services*, was much less prominent in the community maps, ranking only 12^th^ for indegree centrality. Other important outcomes from the literature, *fear and misconception about COVID* and *access to communication modalities*, were less important or absent in stakeholder maps. On the other hand, *increased household conflicts*, identified as the third most prominent outcome category by Bauchi community stakeholders, was not reported at all as an outcome in the studies included in the scoping review.

Table 5 compares literature and stakeholder rankings of outdegree centrality, as an indicator of the importance of categories as causes in the maps. Both the literature and stakeholders identified *lockdown and control measures* and *poverty, hunger, and lack of food* as prominent causes of other categories. However, *fear and misconception about COVID* was a much stronger cause in research studies than among community members. *Access to communication modalities,* prominent as a mitigation of problems in the literature, was not mentioned at all by community stakeholders. *Increased household conflicts* and *reduced education* were much more important causes of problems for community stakeholders than in the literature.

We compared the factors within each of the top five indegree and outdegree centrality categories between the literature and stakeholder maps (see Supplemental Material 7). For most categories, the factors were quite similar between the literature and stakeholder maps. For example, in the category *reduced businesses and jobs*, the common factors included market closures, job and income loss, farm and non-farm closures. The individual factors in the literature tended to be broader than those identified by community stakeholders. Stakeholder maps included factors specific to their context, such as challenges with religious schooling, household conflicts, loss of Hajj deposits, and limited access to farm tools. The literature maps also included some highly specific factors described in studies of particular population groups, such as community health workers, elders, and adolescent students.

## Discussion

This scoping review identified factors associated with negative experiences of the COVID-19 pandemic in rural Nigeria in published literature and juxtaposed the results with stakeholder perspectives from one area of Bauchi State in Northern Nigeria. There was a large overlap in themes, such as poverty, hunger, and lack of food, fear and misconceptions of COVID, as well as the detrimental effects of lockdown and social distancing measures. Both the literature and stakeholders highlighted the critical role of food insecurity, exacerbated by market closures and price hikes. There were also important differences. The literature emphasised reduced health services as an important outcome, while stakeholders identified increased household conflicts as a key outcome. Access to communication modalities was identified as an important influence on pandemic impact in the literature, but did not feature in the stakeholder maps. Stress and mental health issues were important outcomes of the pandemic in both the literature and stakeholder maps; community stakeholders also saw them as causing other problems.

Studies in other countries reported that COVID-19 exacerbated existing challenges in rural communities, including poverty, isolation, socio-economic marginalization, and loss of livelihood and income, even in high income countries such as Canada, US and Australia.^61^ A021 review reported that lockdown measures in six low- and middle-income countries (LMIC), including Nigeria, disproportionately impacted vulnerable populations, who were more at risk from domestic violence, school discontinuity, disruptions in the informal economic sector and limited health system capacity.^62^

Stakeholder maps identified as important some factors that were rarely, if at all, reported in the literature, even though we deliberately restricted the scope of the review to the rural Nigerian setting. It was not that the studies in the review examined the influence of these factors and found them not to be important; rather they did not examine them at all. What gets studied often depends on the interests and biases of researchers, sometimes influenced by the interests and biases of funding bodies. This bias carries over into systematic and scoping reviews, which can only include what is “out there”, even if they attempt to include grey literature. Chackalackal and colleagues broadened the scope of their literature review of the effects of lockdown in LMIC by including mass media reports as well as journal articles.^62^ Contextualising literature findings with local knowledge, as we did in this case, ensures inclusion of locally important causes of a problem and provides evidence to support codesign of locally appropriate solutions. Relying on literature reviews, without local contextualisation, to guide actions may miss important issues.

Our contextualised scoping review confirms the multifaceted impacts of the COVID-19 pandemic in rural Nigeria. By integrating published research with community knowledge, it provides a nuanced understanding of the pandemic’s challenges and opportunities for recovery. As pandemic recovery efforts continue, prioritizing local perspectives will be essential in fostering sustainable and resilient systems in vulnerable communities. This literature review, contextualised by local stakeholder perspectives, was part of a larger project that included a household survey about experiences before and during the pandemic, a process for sharing the findings with local communities, and support for community groups and service providers to plan and implement local actions to support pandemic recovery.

### Limitations

The review was limited to English-language peer-reviewed articles, potentially excluding relevant data from unpublished studies or reports and non-English sources. Nigerian scientific literature is almost all in English, so we do not expect the language restriction introduced an important bias. Excluding grey literature may potentially have missed some relevant reports. Fuzzy cognitive mapping, while robust in integrating diverse data, may oversimplify complex causal relationships. The stakeholder maps were from communities in a single local government area of Bauchi State. The findings from the maps are likely relevant to other nearby states, but we do not suggest they are generalizable to other rural Nigerian contexts or rural contexts in other countries. The purpose of creating the stakeholder maps and using them to contextualise the literature review was to inform *local* action contextualizing the literature to other Nigerian settings would require additional local FCM exercises.

## Conclusions

This scoping review describes important non-clinical impacts of the COVID-19 pandemic in rural Nigeria. Expressing the review findings in a fuzzy cognitive map allowed contextualization with local knowledge in stakeholder fuzzy cognitive maps. The contextualised review contributes to ongoing pandemic recovery efforts tailored to the needs and strengths of rural Nigerian communities.

## Supplemental Material

Supplemental Material - Experience of the COVID-19 Pandemic in Rural Nigeria: A Scoping Review of the Literature Contextualized With Local Knowledge Using Fuzzy Cognitive Mapping Supplemental Material for Experience of the COVID-19 Pandemic in Rural Nigeria: A Scoping Review of the Literature Contextualized With Local Knowledge Using Fuzzy Cognitive Mapping by Mona Z. Ghadirian, Ivan Sarmiento, Natalia Reinoso Chávez, Neil AnderssonAnne Cockcroft in Community Health Equity Research & Policy

Supplemental Material - Experience of the COVID-19 Pandemic in Rural Nigeria: A Scoping Review of the Literature Contextualized With Local Knowledge Using Fuzzy Cognitive Mapping Supplemental Material for Experience of the COVID-19 Pandemic in Rural Nigeria: A Scoping Review of the Literature Contextualized With Local Knowledge Using Fuzzy Cognitive Mapping by Mona Z. Ghadirian, Ivan Sarmiento, Natalia Reinoso Chávez, Neil AnderssonAnne Cockcroft in Community Health Equity Research & Policy

Supplemental Material - Experience of the COVID-19 Pandemic in Rural Nigeria: A Scoping Review of the Literature Contextualized With Local Knowledge Using Fuzzy Cognitive Mapping Supplemental Material for Experience of the COVID-19 Pandemic in Rural Nigeria: A Scoping Review of the Literature Contextualized With Local Knowledge Using Fuzzy Cognitive Mapping by Mona Z. Ghadirian, Ivan Sarmiento, Natalia Reinoso Chávez, Neil AnderssonAnne Cockcroft in Community Health Equity Research & Policy

## Data Availability

The data used in this study are included in the Supplemental Material.
