# Supplementary material for: Experience of the COVID-19 Pandemic in Rural Nigeria: A Scoping Review of the Literature Contextualized With Local Knowledge Using Fuzzy Cognitive Mapping
Source: Community Health Equity Res Policy. 2025 Oct 6;47(2):215–29. doi: 10.1177/2752535X251384511 (PMC13752240; doi:10.1177/2752535X251384511)
Supplement: Supplementary file 1 [file sj-pdf-1-qch-10.1177_2752535X251384511.pdf]

Supplemental material. 3

Guidelines for data extraction.

Fuzzy cognitive mapping from literature reviews of qualitative and quantitative studies.

General procedures

At least two trained researchers will independently extract data using independent forms.

In a group session involving a third reviewer, the team will discuss and resolve any conflicts by consensus. All the identified relationships should be approved at the team meeting.

All reviewers will participate in a pilot extraction using the same set of references to compare results and clarify differences.

Data extraction begins with information about the study and participant characteristics.

Form 1. Characteristics of the studies and participants

Items about the studies:

Authors
Title
Doi
Year of data collection
Study design
Study objectives
Data collection methods
Research setting
Type of participant or community
Sample size
Comments

Items about participants

Are data disaggregated by gender?
Sample by gender
Age range
Occupation

Once Form 1 has been completed for a study, the reviewers should use Form 2 to record all the relationships reported in that study. It can include relationships supported by statistically significant data or by qualitative reports of participant experiences. Reviewers should not include relationships based on reports of assumptions of participants or researchers about these causes.

Form 2 is an edge list (Figure 1) in which the reviewers indicate the factors corresponding to the initiating node (From column) and its outcome (To column). Although fuzzy cognitive maps use weights on a scale to indicate the strength of the relationship, for literature reviews with qualitative and quantitative data, reviewers do not assign weights but only indicate the sign of the relationship. The sign of the relationship will be positive if the increase in the cause results in an increase in the outcome, or negative if the increase in the cause reduces the outcome. For each relationship, reviewers should copy the supporting quote and indicate the page number in the paper.

Figure 1. Generic representation of a fuzzy cognitive map using a graph and an edge list

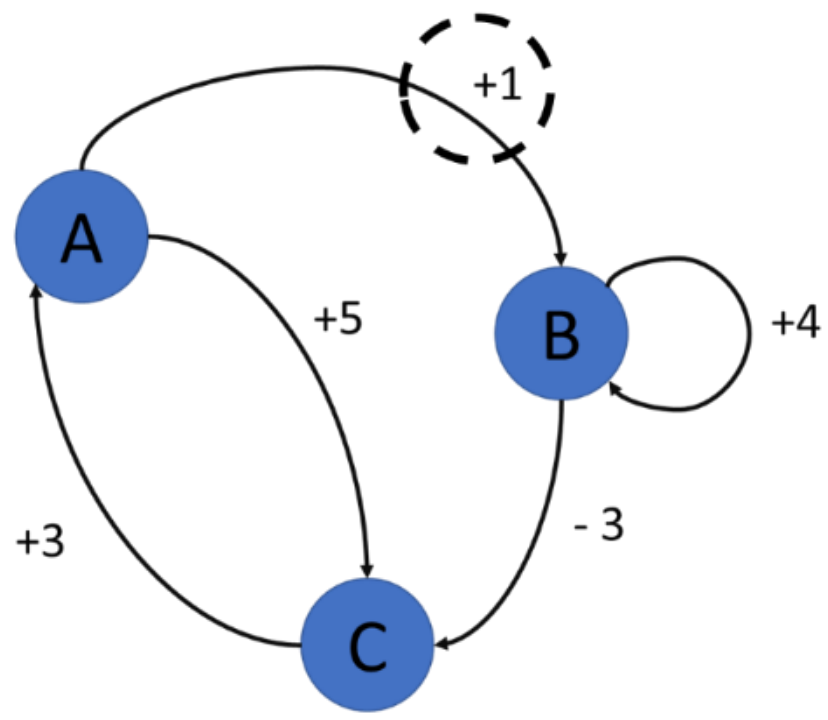

From	To	Weight
A	B	+1
A	C	+5
B	B	+4
B	C	-3
C	A	+3

Legend. The graph on the left contains the same relationships as the edge list on the right. The edge list uses a table format to indicate the nodes involved in the relationship and their weight. Dashed lines indicate the weight of the relationship between node A and node B in both formats.

To identify the relationships, reviewers should follow a stepwise procedure with three moments (Figure 2):

Step 1: Identify the main outcome (Suicidal Behaviours: planning or attempt).

Step 2: Identify direct causes of the main outcome.

Step 3: Identify indirect causes and relationships between factors

Figure 2. Scheme of the steps for identifying the relationships in a study

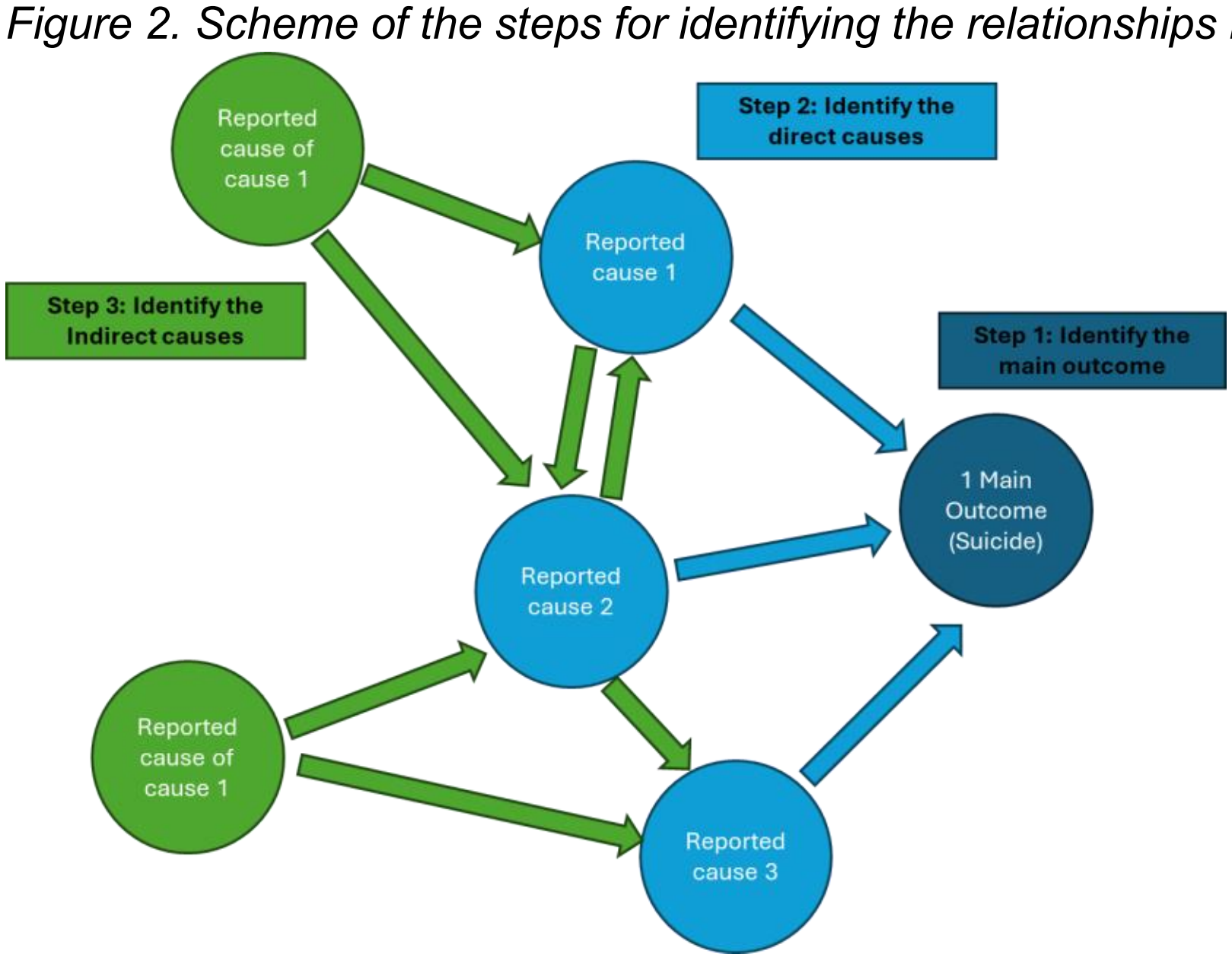

Form 2. Reported impacts of the COVID-19 pandemic

Study	From (factor)	To (factor)	Sign (+/-)	Supporting data	Page

Legend.

From (factor): What contributing factor do the authors propose has a causal effect on suicidal behaviours or other factors?

To (factor): What factor is the outcome or effect?

Sign of the relationship: Positive = Direct relationship, and Negative = Inverse relationship.

Supporting data: Use direct quotes from the article to support each relationship. There may be more than one.

Page: Use the page on the PDF
