# Supplementary material for: Experience of the COVID-19 Pandemic in Rural Nigeria: A Scoping Review of the Literature Contextualized With Local Knowledge Using Fuzzy Cognitive Mapping
Source: Community Health Equity Res Policy. 2025 Oct 6;47(2):215–29. doi: 10.1177/2752535X251384511 (PMC13752240; doi:10.1177/2752535X251384511)
Supplement: Supplementary file 2 [file sj-pdf-2-qch-10.1177_2752535X251384511.pdf]

Supplemental material

Supplemental material 1. PRISMA ScR checklist

SECTION	ITEM	PRISMA-ScR CHECKLIST ITEM	REPORTED ON PAGE #
TITLE			
Title	1	Identify the report as a scoping review.	Title
ABSTRACT			
Structured summary	2	Provide a structured summary that includes (as applicable): background, objectives, eligibility criteria, sources of evidence, charting methods, results, and conclusions that relate to the review questions and objectives.	Page 1
INTRODUCTION			
Rationale	3	Describe the rationale for the review in the context of what is already known. Explain why the review questions/objectives lend themselves to a scoping review approach.	Line 54
Objectives	4	Provide an explicit statement of the questions and objectives being addressed with reference to their key elements (e.g., population or participants, concepts, and context) or other relevant key elements used to conceptualize the review questions and/or objectives.	Lines 54 and 74
METHODS			
Protocol and registration	5	Indicate whether a review protocol exists; state if and where it can be accessed (e.g., a Web address); and if available, provide registration information, including the registration number.	NA
Eligibility criteria	6	Specify characteristics of the sources of evidence used as eligibility criteria (e.g., years considered, language, and publication status), and provide a rationale.	Line 83
Information sources*	7	Describe all information sources in the search (e.g., databases with dates of coverage and contact with authors to identify additional sources), as well as the date the most recent search was executed.	Line 78
Search	8	Present the full electronic search strategy for at least 1 database, including any limits used, such that it could be repeated.	Suppl. Mat. 2
Selection of sources of evidence†	9	State the process for selecting sources of evidence (i.e., screening and eligibility) included in the scoping review.	Line 91
Data charting process‡	10	Describe the methods of charting data from the included sources of evidence (e.g., calibrated forms or forms that have been tested by the team before their use, and whether data charting was done independently or in duplicate) and any processes for obtaining and confirming data from investigators.	Lines 96
Data items	11	List and define all variables for which data were sought and any assumptions and simplifications made.	Lines 101 and 106
Critical appraisal of individual sources of evidence§	12	If done, provide a rationale for conducting a critical appraisal of included sources of evidence; describe the methods used and how this information was used in any data synthesis (if appropriate).	NA
Synthesis of results	13	Describe the methods of handling and summarizing the data that were charted.	Line 122
RESULTS			
Selection of sources of evidence	14	Give numbers of sources of evidence screened, assessed for eligibility, and included in the review, with reasons for exclusions at each stage, ideally using a flow diagram.	Figure 1
Characteristics of sources of evidence	15	For each source of evidence, present characteristics for which data were charted and provide the citations.	Table 2
Critical appraisal	16	If done, present data on critical appraisal of included sources of evidence	NA

SECTION	ITEM	PRISMA-ScR CHECKLIST ITEM	REPORTED ON PAGE #
within sources of evidence		(see item 12).	
Results of individual sources of evidence	17	For each included source of evidence, present the relevant data that were charted that relate to the review questions and objectives.	Suppl. Mat. 3
Synthesis of results	18	Summarize and/or present the charting results as they relate to the review questions and objectives.	Fig. 2, Tables 3, 4, 5 and 6
DISCUSSION			
Summary of evidence	19	Summarize the main results (including an overview of concepts, themes, and types of evidence available), link to the review questions and objectives, and consider the relevance to key groups.	Line 282
Limitations	20	Discuss the limitations of the scoping review process.	Line 316
Conclusions	21	Provide a general interpretation of the results with respect to the review questions and objectives, as well as potential implications and/or next steps.	Line 311
FUNDING			
Funding	22	Describe sources of funding for the included sources of evidence, as well as sources of funding for the scoping review. Describe the role of the funders of the scoping review.	Title page

Supplemental material 2. Search matrices

PubMed

(COVID-19[Mesh] OR "COVID-19 Testing"[Mesh] OR "COVID-19 Vaccines"[Mesh] OR SARS-CoV-2[Mesh] OR (COVID[tw] OR COVID19[tw] OR 2019nCoV[tw] OR nCoV*[tw] OR hCoV*[tw] OR CoV2[tw] OR CoV-2[tw] OR sarscov2[tw] OR sarscov-2[tw] OR SARS-2-CoV[tw] OR SARS-CoV-19[tw] OR Sars-coronavirus2[tw] OR "SARS coronavirus*"[tw] OR "SARS-like coronavirus*"[tw] OR Coronavirus-19[tw] OR (Coronavirus[tw] AND 2019[tw]) OR "Coronavirus 2"[tw] OR ((novel[tw] OR new[tw]) AND (CoV[tw] OR covid*[tw] OR coronavirus*[tw] OR "corona virus*"[tw] OR pandemic*[tw])) OR (coronavirus*[tw] AND pneumonia[tw])) OR coronavirus*[ti] OR corona[ti] OR pandemic*[ti])

AND ("Rural Health Services"[Mesh] OR "Rural Health"[Mesh] OR "Rural Nursing"[Mesh] OR "Suburban Health"[Mesh] OR "Medically Underserved Area"[Mesh] OR "Hospitals, Rural"[Mesh] OR "Rural Population"[Mesh] OR rural*[tw] OR ((remote[tw] OR nonmetropolitan[tw] OR "non metropolitan"[tw] OR suburb*[tw] OR developing[tw] OR "less developed"[tw] OR “lesser developed” OR "under developed"[tw] OR underdeveloped[tw] OR "middle income"[tw] OR "low income"[tw] OR underserved[tw] OR "under served"[tw] OR deprived[tw] OR poor[tw]) AND (communit*[tw] OR area*[tw] OR village*[tw] OR region*[tw] OR province*[tw] OR setting*[tw])) OR ((remote[tw] OR nonmetropolitan[tw] OR "non metropolitan"[tw] OR suburb*[tw] OR "middle income"[tw] OR "low income"[tw] OR underserved[tw] OR "under served"[tw] OR deprived[tw] OR poor[tw] OR village*[tw]) AND population*[tw]) OR ((remote[tw] OR regional[tw] OR nonmetropolitan[tw] OR "non metropolitan"[tw] OR suburb*[tw] OR village*[tw]) AND (clinic[tw] OR clinics[tw] OR hospital[tw] OR hospitals[tw] OR facility[tw] OR facilities[tw] OR "health center*"[tw] OR "health centre*"[tw] OR "health care center*"[tw] OR "health care centre*"[tw] OR "healthcare center*"[tw] OR "healthcare centre*"[tw] OR "medical center*"[tw] OR "medical centre*"[tw])) OR ((shortage[tw] OR understaffed[tw] OR "under staffed"[tw]) AND area*[tw]))

AND ("Nigeria"[mh] OR Nigeria*[all] OR "western Africa"[tw] OR "west Africa"[tw])

Scopus

(INDEXTERMS("COVID-19") OR INDEXTERMS("SARS-CoV-2") OR (TITLE-ABS-KEY("COVID") OR TITLE-ABS-KEY("COVID19") OR TITLE-ABS-KEY("2019nCoV") OR TITLE-ABS-KEY("nCoV*") OR TITLE-ABS-KEY("hCoV*") OR TITLE-ABS-KEY("CoV2") OR TITLE-ABS-KEY("CoV-2") OR TITLE-ABS-KEY("sarscov2") OR TITLE-ABS-KEY("sarscov-2") OR TITLE-ABS-KEY("SARS-2-CoV") OR TITLE-ABS-KEY("SARS-CoV-19") OR TITLE-ABS-KEY("Sars-coronavirus2") OR TITLE-ABS-KEY("SARS coronavirus*") OR TITLE-ABS-KEY("SARS-like coronavirus*") OR TITLE-ABS-KEY("Coronavirus-19") OR (TITLE-ABS-KEY("Coronavirus") AND TITLE-ABS-KEY("2019")) OR TITLE-ABS-KEY("Coronavirus 2") OR ((TITLE-ABS-KEY("novel") OR TITLE-ABS-

KEY("new")) AND (TITLE-ABS-KEY("CoV") OR TITLE-ABS-KEY("covid*") OR TITLE-ABS-KEY("coronavirus*") OR TITLE-ABS-KEY("corona virus*") OR TITLE-ABS-KEY("pandemic*"))) OR (TITLE-ABS-KEY("coronavirus*") AND TITLE-ABS-KEY("pneumonia"))) OR TITLE("coronavirus*") OR TITLE("corona") OR TITLE("pandemic*"))

AND (INDEXTERMS("Rural Health Services") OR INDEXTERMS("Rural Health") OR INDEXTERMS("Rural Nursing") OR INDEXTERMS("Suburban Health") OR INDEXTERMS("Medically Underserved Area") OR INDEXTERMS("Hospitals, Rural") OR INDEXTERMS("Rural Population") OR TITLE-ABS-KEY("rural*") OR ((TITLE-ABS-KEY("remote") OR TITLE-ABS-KEY("nonmetropolitan") OR TITLE-ABS-KEY("non metropolitan") OR TITLE-ABS-KEY("suburb*") OR TITLE-ABS-KEY("developing") OR TITLE-ABS-KEY("less developed") OR TITLE-ABS-KEY("lesser developed") OR TITLE-ABS-KEY("under developed") OR TITLE-ABS-KEY("underdeveloped") OR TITLE-ABS-KEY("middle income") OR TITLE-ABS-KEY("low income") OR TITLE-ABS-KEY("underserved") OR TITLE-ABS-KEY("under served") OR TITLE-ABS-KEY("deprived") OR TITLE-ABS-KEY("poor")) AND (TITLE-ABS-KEY("communit*") OR TITLE-ABS-KEY("area*") OR TITLE-ABS-KEY("village*") OR TITLE-ABS-KEY("region*") OR TITLE-ABS-KEY("province*") OR TITLE-ABS-KEY("setting*"))) OR ((TITLE-ABS-KEY("remote") OR TITLE-ABS-KEY("nonmetropolitan") OR TITLE-ABS-KEY("non metropolitan") OR TITLE-ABS-KEY("suburb*") OR TITLE-ABS-KEY("middle income") OR TITLE-ABS-KEY("low income") OR TITLE-ABS-KEY("underserved") OR TITLE-ABS-KEY("under served") OR TITLE-ABS-KEY("deprived") OR TITLE-ABS-KEY("poor") OR TITLE-ABS-KEY("village*")) AND TITLE-ABS-KEY("population*")) OR ((TITLE-ABS-KEY("remote") OR TITLE-ABS-KEY("regional") OR TITLE-ABS-KEY("nonmetropolitan") OR TITLE-ABS-KEY("non metropolitan") OR TITLE-ABS-KEY("suburb*") OR TITLE-ABS-KEY("village*"))))

AND (INDEXTERMS("Nigeria") OR "Nigeria*" OR TITLE-ABS-KEY("western Africa") OR TITLE-ABS-KEY("west Africa"))

CINAHL

((MH "COVID-19+") OR (MH "COVID-19 Testing+") OR (MH "COVID-19 Vaccines+") OR (MH "SARS-CoV-2+") OR (COVID OR COVID19 OR 2019nCoV OR nCoV* OR hCoV* OR CoV2 OR CoV-2 OR sarscov2 OR sarscov-2 OR SARS-2-CoV OR SARS-CoV-19 OR Sars-coronavirus2 OR "SARS coronavirus*" OR "SARS-like coronavirus*" OR Coronavirus-19 OR (Coronavirus AND 2019) OR "Coronavirus 2" OR ((novel OR new) AND (CoV OR covid* OR coronavirus* OR "corona virus*" OR pandemic*)) OR (coronavirus* AND pneumonia)) OR TI coronavirus* OR TI corona OR TI pandemic*)

((MH "Rural Health Services+") OR (MH "Rural Health+") OR (MH "Rural Nursing+") OR (MH "Suburban Health+") OR (MH "Medically Underserved Area+") OR (MH "Hospitals, Rural+") OR (MH "Rural

Population+") OR rural* OR ((remote OR nonmetropolitan OR "non metropolitan" OR suburb* OR developing OR "less developed" OR "lesser developed" OR "under developed" OR underdeveloped OR "middle income" OR "low income" OR underserved OR "under served" OR deprived OR poor) AND (communit* OR area* OR village* OR region* OR province* OR setting*)) OR ((remote OR nonmetropolitan OR "non metropolitan" OR suburb* OR "middle income" OR "low income" OR underserved OR "under served" OR deprived OR poor OR village*) AND population*) OR ((remote OR regional OR nonmetropolitan OR "non metropolitan" OR suburb* OR village*) AND (clinic OR clinics OR hospital OR hospitals OR facility OR facilities OR "health center*" OR "health centre*" OR "health care center*" OR "health care centre*" OR "healthcare center*" OR "healthcare centre*" OR "medical center*" OR "medical centre*")) OR ((shortage OR understaffed OR "under staffed") AND area*)) ((MH "Nigeria+") OR Nigeria* OR "western Africa" OR "west Africa")

Supplemental material 3. Guidelines for data extraction. Fuzzy cognitive mapping from literature reviews of qualitative and quantitative studies.

File: R1_COVID_Lit_Suppl 3.docx

Supplemental material 4. Table of relationships and references considered in fuzzy cognitive mapping of the literature

**Relationship**[1]	**References**[2]
F183-F33	#294 - Nnama-Okechukwu 2020, #354 - Osarenkhoe 2023, #358 - Owhonda 2022
F199-F84	#148 - Ekoh 2020, #150 - Ekoh 2021, #478 - Ekoh 2022
F68-F151	#148 - Ekoh 2020, #150 - Ekoh 2021, #478 - Ekoh 2022
F82-F151	#148 - Ekoh 2020, #150 - Ekoh 2021, #478 - Ekoh 2022
F82-F198	#148 - Ekoh 2020, #150 - Ekoh 2021, #478 - Ekoh 2022
F136-F198	#150 - Ekoh 2021, #478 - Ekoh 2022
F144-F198	#148 - Ekoh 2020, #150 - Ekoh 2021
F145-F35	#213 - Iliyasu 2023, #478 - Ekoh 2022
F156-F198	#148 - Ekoh 2020, #150 - Ekoh 2021
F183-F61	#321 - Ojo 2023, #354 - Osarenkhoe 2023
F22-F223	#361 - Oyekale 2022, #443 - Wang 2022
F58-F101	#222 - Josephson 2021, #266 - Maredia 2022
F68-F198	#150 - Ekoh 2021, #478 - Ekoh 2022
F68-F82	#148 - Ekoh 2020, #150 - Ekoh 2021
F82-F136	#150 - Ekoh 2021, #478 - Ekoh 2022
F90-F158	#450 - Yahemba 2023, #696 - Reghagwa 2021
F92-F151	#148 - Ekoh 2020, #478 - Ekoh 2022
F95-F223	#377 - Rasaq 2023, #443 - Wang 2022
F02-F44	#450 - Yahemba 2023
F03-F08	#450 - Yahemba 2023
F03-F128	#450 - Yahemba 2023
F03-F141	#450 - Yahemba 2023

F03-F45	#450 - Yahemba 2023
F05-F196	#337 - Olanrewaju 2021
F07-F06	#337 - Olanrewaju 2021
F09-F63	#150 - Ekoh 2021
F09-F78	#150 - Ekoh 2021
F09-F84	#150 - Ekoh 2021
F100-F109	#450 - Yahemba 2023
F106-F221	#213 - Iliyasu 2023
F108-F134	#280 - Mueller 2023
F109-F30	#213 - Iliyasu 2023
F110-F221	#213 - Iliyasu 2023
F111-F221	#213 - Iliyasu 2023
F112-F221	#213 - Iliyasu 2023
F113-F221	#213 - Iliyasu 2023
F113-F223	#377 - Rasaq 2023
F114-F221	#213 - Iliyasu 2023
F115-F221	#213 - Iliyasu 2023
F116-F68	#266 - Maredia 2022
F118-F221	#213 - Iliyasu 2023
F11-F63	#150 - Ekoh 2021
F11-F72	#150 - Ekoh 2021
F120-F221	#213 - Iliyasu 2023
F121-F33	#317 - Ojeka-John 2023
F121-F34	#317 - Ojeka-John 2023
F122-F73	#696 - Reghagwa 2021
F124-F01	#450 - Yahemba 2023
F124-F10	#47 - Ajefu 2023

F124-F101	#450 - Yahemba 2023
F124-F126	#450 - Yahemba 2023
F124-F25	#450 - Yahemba 2023
F125-F03	#47 - Ajefu 2023
F125-F10	#47 - Ajefu 2023
F125-F13	#47 - Ajefu 2023
F127-F223	#377 - Rasaq 2023
F128-F09	#150 - Ekoh 2021
F128-F136	#150 - Ekoh 2021
F128-F198	#150 - Ekoh 2021
F128-F63	#150 - Ekoh 2021
F128-F78	#150 - Ekoh 2021
F128-F84	#150 - Ekoh 2021
F129-F33	#163 - Ezeah 2020
F131-F81	#148 - Ekoh 2020
F133-F13	#47 - Ajefu 2023
F135-F180	#280 - Mueller 2023
F136-F151	#478 - Ekoh 2022
F137-F06	#337 - Olanrewaju 2021
F138-F06	#337 - Olanrewaju 2021
F138-F09	#150 - Ekoh 2021
F138-F63	#150 - Ekoh 2021
F138-F78	#150 - Ekoh 2021
F138-F84	#150 - Ekoh 2021
F139-F06	#337 - Olanrewaju 2021
F140-F06	#337 - Olanrewaju 2021
F141-F08	#450 - Yahemba 2023

F141-F221	#213 - Iliyasu 2023
F141-F45	#450 - Yahemba 2023
F142-F06	#337 - Olanrewaju 2021
F142-F63	#150 - Ekoh 2021
F143-F86	#148 - Ekoh 2020
F144-F03	#450 - Yahemba 2023
F144-F08	#450 - Yahemba 2023
F144-F103	#294 - Nnama-Okechukwu 2020
F144-F128	#450 - Yahemba 2023
F144-F141	#450 - Yahemba 2023
F144-F15	#450 - Yahemba 2023
F144-F150	#450 - Yahemba 2023
F144-F164	#450 - Yahemba 2023
F144-F172	#450 - Yahemba 2023
F144-F194	#450 - Yahemba 2023
F144-F41	#450 - Yahemba 2023
F144-F46	#450 - Yahemba 2023
F144-F67	#450 - Yahemba 2023
F144-F82	#148 - Ekoh 2020
F144-F97	#450 - Yahemba 2023
F144-F98	#450 - Yahemba 2023
F144-F99	#450 - Yahemba 2023
F145-F117	#213 - Iliyasu 2023
F145-F151	#478 - Ekoh 2022
F145-F161	#478 - Ekoh 2022
F145-F163	#294 - Nnama-Okechukwu 2020
F145-F175	#149 - Ekoh 2021

F145-F198	#478 - Ekoh 2022
F145-F200	#478 - Ekoh 2022
F145-F226	#213 - Iliyasu 2023
F145-F227	#294 - Nnama-Okechukwu 2020
F145-F40	#213 - Iliyasu 2023
F145-F67	#149 - Ekoh 2021
F145-F68	#149 - Ekoh 2021
F145-F74	#696 - Reghagwa 2021
F147-F68	#266 - Maredia 2022
F148-F179	#149 - Ekoh 2021
F149-F195	#213 - Iliyasu 2023
F149-F225	#213 - Iliyasu 2023
F14-F221	#213 - Iliyasu 2023
F151-F81	#148 - Ekoh 2020
F152-F223	#377 - Rasaq 2023
F153-F15	#450 - Yahemba 2023
F153-F172	#450 - Yahemba 2023
F153-F215	#450 - Yahemba 2023
F153-F46	#450 - Yahemba 2023
F153-F99	#450 - Yahemba 2023
F154-F02	#450 - Yahemba 2023
F154-F44	#450 - Yahemba 2023
F155-F113	#266 - Maredia 2022
F155-F68	#266 - Maredia 2022
F156-F151	#148 - Ekoh 2020
F156-F73	#696 - Reghagwa 2021
F156-F81	#148 - Ekoh 2020

F156-F82	#148 - Ekoh 2020
F157-F13	#47 - Ajefu 2023
F158-F15	#450 - Yahemba 2023
F158-F170	#450 - Yahemba 2023
F158-F172	#450 - Yahemba 2023
F158-F46	#450 - Yahemba 2023
F158-F51	#450 - Yahemba 2023
F158-F73	#696 - Reghagwa 2021
F158-F99	#450 - Yahemba 2023
F159-F223	#377 - Rasaq 2023
F15-F99	#450 - Yahemba 2023
F160-F172	#478 - Ekoh 2022
F161-F200	#478 - Ekoh 2022
F162-F221	#213 - Iliyasu 2023
F165-F223	#377 - Rasaq 2023
F166-F223	#377 - Rasaq 2023
F167-F168	#149 - Ekoh 2021
F167-F179	#149 - Ekoh 2021
F167-F209	#149 - Ekoh 2021
F167-F26	#149 - Ekoh 2021
F167-F27	#149 - Ekoh 2021
F167-F54	#149 - Ekoh 2021
F168-F104	#450 - Yahemba 2023
F168-F172	#450 - Yahemba 2023
F168-F179	#149 - Ekoh 2021
F168-F221	#213 - Iliyasu 2023
F170-F51	#450 - Yahemba 2023

F171-F221	#213 - Iliyasu 2023
F173-F215	#317 - Ojeka-John 2023
F174-F34	#294 - Nnama-Okechukwu 2020
F177-F38	#478 - Ekoh 2022
F178-F223	#377 - Rasaq 2023
F182-F132	#348 - Onukwuli 2023
F182-F172	#442 - Wang 2023
F182-F23	#442 - Wang 2023
F182-F62	#442 - Wang 2023
F183-F08	#81 - Assefa 2023
F183-F102	#385 - Rudin-Rush 2022
F183-F105	#358 - Owhonda 2022
F183-F107	#362 - Oyekale 2023
F183-F119	#266 - Maredia 2022
F183-F123	#266 - Maredia 2022
F183-F130	#288 - Ndubuisi-Okoroezi 2022
F183-F172	#81 - Assefa 2023
F183-F216	#273 - Michael 2021
F183-F217	#72 - Amuzie 2023
F183-F34	#294 - Nnama-Okechukwu 2020
F184-F168	#347 - Omoronyia 2020
F184-F33	#347 - Omoronyia 2020
F184-F42	#450 - Yahemba 2023
F184-F92	#450 - Yahemba 2023
F185-F151	#478 - Ekoh 2022
F185-F176	#478 - Ekoh 2022
F186-F134	#280 - Mueller 2023

F187-F134	#280 - Mueller 2023
F188-F06	#337 - Olanrewaju 2021
F189-F06	#337 - Olanrewaju 2021
F190-F06	#337 - Olanrewaju 2021
F191-F134	#280 - Mueller 2023
F192-F134	#280 - Mueller 2023
F193-F135	#280 - Mueller 2023
F193-F180	#280 - Mueller 2023
F193-F222	#280 - Mueller 2023
F194-F18	#731 - Wang 2021
F194-F21	#731 - Wang 2021
F194-F23	#731 - Wang 2021
F194-F45	#450 - Yahemba 2023
F194-F97	#450 - Yahemba 2023
F194-F98	#450 - Yahemba 2023
F196-F05	#150 - Ekoh 2021
F196-F63	#150 - Ekoh 2021
F197-F78	#148 - Ekoh 2020
F198-F151	#478 - Ekoh 2022
F199-F131	#148 - Ekoh 2020
F199-F151	#478 - Ekoh 2022
F199-F198	#148 - Ekoh 2020
F199-F78	#150 - Ekoh 2021
F199-F81	#148 - Ekoh 2020
F201-F198	#150 - Ekoh 2021
F202-F06	#337 - Olanrewaju 2021
F203-F06	#337 - Olanrewaju 2021

F204-F221	#213 - Iliyasu 2023
F205-F73	#696 - Reghagwa 2021
F206-F105	#294 - Nnama-Okechukwu 2020
F208-F207	#149 - Ekoh 2021
F209-F54	#149 - Ekoh 2021
F210-F34	#149 - Ekoh 2021
F211-F68	#149 - Ekoh 2021
F212-F168	#149 - Ekoh 2021
F212-F179	#149 - Ekoh 2021
F213-F210	#149 - Ekoh 2021
F213-F211	#149 - Ekoh 2021
F213-F34	#149 - Ekoh 2021
F213-F68	#149 - Ekoh 2021
F214-F06	#337 - Olanrewaju 2021
F218-F221	#213 - Iliyasu 2023
F222-F180	#280 - Mueller 2023
F224-F168	#58 - Akwaowo 2021
F224-F172	#478 - Ekoh 2022
F227-F163	#294 - Nnama-Okechukwu 2020
F25-F101	#450 - Yahemba 2023
F28-F08	#213 - Iliyasu 2023
F28-F117	#213 - Iliyasu 2023
F28-F15	#450 - Yahemba 2023
F28-F172	#450 - Yahemba 2023
F28-F46	#450 - Yahemba 2023
F28-F99	#450 - Yahemba 2023
F29-F16	#450 - Yahemba 2023

F29-F172	#450 - Yahemba 2023
F31-F225	#213 - Iliyasu 2023
F32-F02	#450 - Yahemba 2023
F32-F104	#450 - Yahemba 2023
F32-F154	#450 - Yahemba 2023
F32-F168	#450 - Yahemba 2023
F32-F172	#450 - Yahemba 2023
F32-F44	#450 - Yahemba 2023
F33-F223	#377 - Rasaq 2023
F35-F161	#478 - Ekoh 2022
F35-F200	#478 - Ekoh 2022
F35-F40	#213 - Iliyasu 2023
F35-F78	#150 - Ekoh 2021
F36-F04	#47 - Ajefu 2023
F36-F10	#47 - Ajefu 2023
F36-F12	#47 - Ajefu 2023
F36-F13	#47 - Ajefu 2023
F37-F223	#397 - Sato 2022
F41-F164	#450 - Yahemba 2023
F43-F172	#450 - Yahemba 2023
F46-F15	#450 - Yahemba 2023
F46-F172	#450 - Yahemba 2023
F46-F99	#450 - Yahemba 2023
F48-F197	#478 - Ekoh 2022
F49-F101	#450 - Yahemba 2023
F49-F25	#450 - Yahemba 2023
F50-F104	#450 - Yahemba 2023

F50-F168	#450 - Yahemba 2023
F50-F172	#450 - Yahemba 2023
F50-F34	#294 - Nnama-Okechukwu 2020
F52-F168	#294 - Nnama-Okechukwu 2020
F53-F131	#148 - Ekoh 2020
F53-F81	#148 - Ekoh 2020
F55-F143	#148 - Ekoh 2020
F55-F86	#148 - Ekoh 2020
F56-F215	#317 - Ojeka-John 2023
F57-F129	#163 - Ezeah 2020
F57-F168	#163 - Ezeah 2020
F57-F33	#163 - Ezeah 2020
F58-F08	#81 - Assefa 2023
F58-F113	#222 - Josephson 2021
F58-F123	#266 - Maredia 2022
F58-F146	#266 - Maredia 2022
F58-F169	#450 - Yahemba 2023
F58-F17	#731 - Wang 2021
F58-F18	#731 - Wang 2021
F58-F181	#731 - Wang 2021
F58-F19	#731 - Wang 2021
F58-F194	#731 - Wang 2021
F58-F197	#478 - Ekoh 2022
F58-F20	#731 - Wang 2021
F58-F21	#731 - Wang 2021
F58-F23	#731 - Wang 2021
F58-F24	#731 - Wang 2021

F58-F27	#450 - Yahemba 2023
F58-F47	#450 - Yahemba 2023
F58-F48	#478 - Ekoh 2022
F58-F87	#222 - Josephson 2021
F59-F61	#377 - Rasaq 2023
F60-F223	#377 - Rasaq 2023
F64-F209	#149 - Ekoh 2021
F64-F54	#149 - Ekoh 2021
F65-F223	#377 - Rasaq 2023
F65-F33	#294 - Nnama-Okechukwu 2020
F65-F37	#397 - Sato 2022
F66-F151	#478 - Ekoh 2022
F66-F198	#478 - Ekoh 2022
F66-F94	#478 - Ekoh 2022
F67-F113	#213 - Iliyasu 2023
F67-F221	#213 - Iliyasu 2023
F67-F68	#149 - Ekoh 2021
F68-F136	#150 - Ekoh 2021
F68-F78	#150 - Ekoh 2021
F68-F81	#148 - Ekoh 2020
F68-F84	#150 - Ekoh 2021
F69-F73	#696 - Reghagwa 2021
F70-F69	#696 - Reghagwa 2021
F70-F73	#696 - Reghagwa 2021
F71-F69	#696 - Reghagwa 2021
F71-F73	#696 - Reghagwa 2021
F75-F172	#150 - Ekoh 2021

F76-F80	#696 - Reghagwa 2021
F77-F200	#478 - Ekoh 2022
F77-F78	#478 - Ekoh 2022
F79-F73	#696 - Reghagwa 2021
F82-F81	#148 - Ekoh 2020
F83-F63	#150 - Ekoh 2021
F83-F72	#150 - Ekoh 2021
F84-F151	#478 - Ekoh 2022
F84-F73	#696 - Reghagwa 2021
F84-F78	#150 - Ekoh 2021
F85-F78	#478 - Ekoh 2022
F87-F05	#150 - Ekoh 2021
F87-F196	#150 - Ekoh 2021
F87-F63	#150 - Ekoh 2021
F88-F04	#47 - Ajefu 2023
F88-F10	#47 - Ajefu 2023
F88-F13	#47 - Ajefu 2023
F89-F08	#450 - Yahemba 2023
F89-F15	#450 - Yahemba 2023
F89-F158	#450 - Yahemba 2023
F89-F170	#450 - Yahemba 2023
F89-F219	#450 - Yahemba 2023
F89-F46	#450 - Yahemba 2023
F89-F51	#450 - Yahemba 2023
F89-F99	#450 - Yahemba 2023
F90-F15	#450 - Yahemba 2023
F90-F170	#450 - Yahemba 2023

F90-F172	#450 - Yahemba 2023
F90-F221	#213 - Iliyasu 2023
F90-F46	#450 - Yahemba 2023
F90-F51	#450 - Yahemba 2023
F90-F73	#696 - Reghagwa 2021
F90-F99	#450 - Yahemba 2023
F91-F221	#213 - Iliyasu 2023
F92-F168	#58 - Akwaowo 2021
F92-F81	#148 - Ekoh 2020
F92-F84	#478 - Ekoh 2022
F93-F220	#213 - Iliyasu 2023
F94-F151	#478 - Ekoh 2022
F94-F198	#478 - Ekoh 2022
F96-F39	#450 - Yahemba 2023

[1] A legend of all the factors are found in Supplemental material 5.

[2] The articles referenced are found below

Supplemental material 5. COVID impact factors identified in the studies and their grouping into 29 broader categories

Factor Code		Standard factor label	Category	Category Code
F37		Belief that vaccine will not work	Fear and misconceptions about COVID	C01
F53		Concern for safety/ fear of infecting older people		
F64		Disbelief about the existence of COVID-19		
F65		Distrust in government, health organizations, and medical doctors		
F66		Early predictions about the pandemic in SSA		
F76		Elders fear of getting infected		
F89		Fear and stigma of others thinking you are COVID positive		
F90		Fear of contracting COVID-19 at the health facility		
F91		Fear of contracting COVID-19 in transportation		
F92		Fear of getting infected		
F95		Fear of vaccine safety/ side effects		
F94		Fear of infecting older parents		
F122		Inadequate support from government to boost access to health information		
F154		Misinformation about COVID-19		
F179		Perceived risk of outbreak		
F205		Too many conflictive publications about COVID-19		
F32	*	Awareness campaigns		
F33	*	Awareness/ knowledge of COVID-19		
F34	*	Awareness/ knowledge of COVID-19 preventive measures		
F56	*	COVID-19 communication in native language (ex. Yoruba)		
F57	*	COVID-19 information source credibility		
F129	*	Information on COVID-19 through interpersonal communication		
F153	*	Ministry of health educational material		
F165	*	Perception of severity of COVID-19		
F166	*	Perceived susceptibility to COVID-19 infection		
F173	*	Relatable COVID communication/information		
F215	*	Understanding COVID-19 information/ communication		
F155		More stringent and prolonged COVID-19 restriction policies	Lockdown and control measures	C02
F58		COVID-19 restrictions / mitigation strategies		
F145		Lockdown and movement restrictions		
F201		State borders closed after COVID		
F156		Movement restrictions		
F144		Lockdown		
F141		Lack of transport		
F01		Difficulty in storing food as advised	Hunger and lack of food	C03
F36		Being sick / injured		
F38		Challenged by reduced economic support (cultural expectations)		
F68		Economic impact of the pandemic		
F81		Elders risk of poverty		
F82		Elders' children financial struggles		
F102		Food insecurity		
F119		Household skipped meals because of lack of food		

F128		Individual lack of money		
F136		Lack funds for transportation		
F138		Lack of electricity		
F167		Poverty		
F176		Retired elders’ dependency on intergenerational support for financial security		
F185		Rural elders not being beneficiaries of pension scheme		
F188		Rural students’ financial constraints		
F195		Shortage of food supply		
F13	*	Access to staple food		
F19	*	Adolescent food consumption		
F25	*	Affordability of food (ability to afford food for their families)		
F26	*	Affordability of preventive tools (masks, soap)		
F101	*	Food consumption		
F107	*	Have health insurance		
F113	*	Higher-income		
F181	*	Rural adolescent consumption of animal sourced food		
F05		Access to digital information and entertainment		
F07		Access to digital technology		
F09		Access to means of communication (smartphones)		
F11		Access to radio and non-digital technology		
F130		Information seeking about COVID		
F135		Knowledge acquired through migrant networks		
F196		Skills/digital literacy in using new digital technologies		
F69		Elder information seeking (mass-media, family members, friends, physicians)	Access to communication modalities	C04
F70		Elder information seeking NOT related to COVID		
F71		Elder information seeking related to COVID		
F73		Elders access to health information		
F85		Elders' use of digital technology		
F63	*	Digital divide/exclusion of elders		
F79	*	Elders’ low health literacy		
F83	*	Elders' lack of interest in new digital technology		
F142	*	Limited internet		
F45		CHW unable to provide routine care		
F51		Compromised immunization in the community		
F93		Fear of healthcare workers harassment during labour		
F100		Focus on COVID-19 in health facilities		
F106		Harassment by security personnel to patients		
F109		Health services (and medical training) focused on COVID (instead of other health conditions)		
F115		Higher parity mothers		
F117		Home birth		
F158		Not visiting health facility	Reduced health services	C05
F162		Overcrowding in clinics		
F204		The chairs and examination beds are not clean		
F216		Unmet needs for family planning		
F217		Unmet needs for family planning among women in extended postpartum period		
F218		Unsure of availability of services		
F02	*	Ability of CHW to provide care		
F08	*	Access to health services		
F10	*	Access to medicine / medical treatment		

F14	*	Access to water, soap, and sanitizer in the health facilities		
F28	*	Availability of health professionals		
F30	*	Availability of health workers for childbirth		
F31	*	Availability of maternal health services		
F74	*	Elders access to physician		
F110	*	Health services utilization before COVID-19		
F150	*	Mass administration of medical campaigns to control Neglected Tropical Diseases		
F170	*	Providing immunization		
F171	*	Proximity to health facility		
F219	*	Use of medicines		
F220	*	Utilization of Antenatal Care		
F221	*	Utilization of maternal health services during the COVID-19 restrictions		
F52		Concern about income loss	Reduced businesses and jobs	C06
F88		Farming disruption		
F116		Highly interdependent/ interconnected rural-urban linkages		
F118		Homemaker occupation		
F123		Income loss		
F133		Job loss		
F146		Loss in income source		
F149		Markets/shops closed		
F157		Non-farm business closure		
F67	*	Economic activities		
F120	*	Husband employment		
F147	*	Market diversity		
F148	*	Market structure		
F175	*	Remittances		
F15		Accumulated undone home chores - Community health workers	Difficulties for community health workers	C07
F16		Added cost for health workers		
F42		CHW distance from their families		
F43		CHW exposure to COVID-19 cases		
F46		CHW workload		
F47		CHW's personal loss and bereavement		
F96		Female CHW		
F99		Female CHW's lack of rest		
F111		Health workers do not use COVID-19 protective equipment		
F140		CHW Lack of time		
F29	*	Availability of health worker resources (transport, personal protective equipment, lights, lab equipment)		
F49	*	Community health volunteers		
F104	*	Good relationships between CHW and the community		
F184	*	Rural community health workers		
F50		Community leaders involved in COVID-19 prevention measures	Traditional rulers' protective role	C08
F207		Traditional ruler as a role model facing COVID-19		
F208		Traditional rulers' preventive practices		
F209		Traditional rulers' ability to ensure community safety		
F210		Traditional rulers creating COVID-19 awareness		
F211		Traditional rulers distribute relief materials (originally from gov't)		
F212		Traditional rulers encouraged protective measures in		

		ceremonies		
F213		Traditional rulers’ protective role in the community		
F183		Rural communities	Rural communities/population	C09
F23		Adolescents not getting any education	Reduced education	C10
F62		Difficulty catching up with education		
F137		Lack of e-learning platforms		
F139		Lack of parental support for rural students		
F189		Rural students’ lack of skills/digital literacy		
F190		Rural students’ preference for face-to-face learning		
F194		School closures		
F214		Unavailability of teachers to support students in the use of e-learning		
F18	*	Adolescent education from other channels		
F21	*	Adolescent self-assessed ability to learn		
F06	*	Access to digital learning among rural students		
F164	*	Parents teaching children alternative skills		
F202	*	Student awareness of digital learning intervention by government		
F203	*	Student learning via radio and television programmes		
F77		Elders living alone (who do not live with either their spouse, children, or grandchildren)	Reduced social contact and activities	C11
F84		Elders' limited contact with other relatives and friends		
F160		Older women living alone (who do not live with either their spouse, children, or grandchildren)		
F200		Social isolation of elders		
F17	*	Adolescent communication with family and friends outside of household		
F27	*	Attend social gatherings (worship, visit family)		
F40	*	Childbirth cultural practices		
F75	*	Elders busy with home and farming		
F80	*	Elders’ movement		
F131	*	Intangible assistance for elders		
F161	*	Out-of-home relationship		
F197	*	Social contact		
F198	*	Social contact between older adults and urban-dwelling children		
F125		Increase in price farming inputs	Price hike	C12
F124		Increase in food prices		
F169		Price of goods		
F35		Ban on social gatherings	Enforced social distance	C13
F199		Social distancing		
F112		Higher husband educational level	Level of schooling	C14
F114		Higher maternal education level		
F187		Rural men secondary education		
F191		Rural women higher education		
F127		Increased schooling level		
F121		Illiterate rural residents		
F168		Preventive health behaviour (e.g. masks)	Preventive behaviours (masks and handwashing)	C15
F105		Hand washing (preventive measure)		
F206		Customary practice (handwashing)		
F04		Access to cleaning supplies		
F12		Access to soap		

F159		Older age	Older age	C16
F177		Retired older men		
F178		Retiree		
F186		Rural men over 65 years		
F22		Adolescents	Adolescents	C17
F182		Rural adolescent students		
F192		Rural women migrating within the same state	Migration within states	C18
F193		Rural women migration		
F180		Risks associated with moving across locations (migration)		
F143		Limited palliatives in rural settings	Reduced Government support	C19
F86	*	Elders' access to palliatives		
F55	*	Consideration for elders' unique nutritional and medical needs		
F224		Women	Woman	C20
F152	*	Men		
F03	*	Access to bank/ATM	Lack of access to money	C21
F163		Parents knowing their children better	Children staying at home	C22
F24		Adolescents stay home more often		
F227		Youth/children staying at home		
F108		Having children		
F59		COVID-19 vaccine availability	COVID-19 Vaccination	C23
F61		COVID-19 vaccine uptake		
F223		Willingness to take COVID vaccine		
F60	*	COVID-19 vaccine cost for the recipient (vaccine not free)		
F41		CHW concern about their children's education	Stress and mental health problems	C24
F72		Elders feeling left out/excluded		
F78		Elders' loneliness / feeling isolated		
F97		Female CHW bringing children to work – stress at work		
F98		Female CHW parental distress		
F172		Psychological distress		
F225		Women are anxious about their childbirth		
F226		Women loneliness in childbirth		
F44	*	CHW pride		
F48		Closure of religious institutions	Reduced religious activities	C25
F54		Confidence in spiritual protection	Religious beliefs	C26
F174		Religious beliefs and practices		
F20		Adolescent media consumption	Increased social vices	C27
F126		Increased crime		
F132		Internet addiction		
F134		Know someone with COVID-19	Know someone with COVID-19	C28
F103		Gender roles redefinition	Change in gender roles	C29
F39	*	Challenges balancing gendered roles as mothers and wives with work		

The table lists the standard name of each factor identified in the scoping review and shows the factors grouped by category. Column one contains the unique codes for factors (F01–F227) and column 5 has the category codes (C01–C29). When calculating indegree and outdegree centrality of each category, the factors marked with an asterisk in column 2 changed their sign to match the sense of the category. For

example, "social contact" (protective) became "lack of social contact" (risk) to match with the overall category of “Reduced social contact and activities”.

Supplemental material 6. Adjacency matrix with all the category relationships identified in the scoping review

See attached Excel file: litRev_Covid_SM_5.xlsx

This adjacency matrix is a tabular representation of the map. Each cell represents a relationship, and the value corresponds to its weight. The relationship goes from the category in the row to the category in the column. At the margins of the matrix, we indicate the centrality measures for each category and its corresponding rank. The names corresponding to each category code and the factors included in each category are shown in Supplemental material 4. Table 3 in the main article includes a brief narrative description of the contents of each category.

Supplemental material 7. Similarities and differences between prominent literature and stakeholder factors

Category	Literature Factors	Stakeholder Factors	Similarities	Differences
**Reduced health services**	CHW unable to provide routine care<br>Compromised immunization in the community<br>Fear of healthcare workers harassment during labour<br>Focus on COVID-19 in health facilities<br>Harassment by security personnel to patients<br>Health services (and medical training) focused on COVID (instead of other health conditions)<br>Higher parity mothers<br>Home birth<br>Not visiting health facility<br>Overcrowding in clinics<br>The chairs and examination beds are not clean	Cannot access maternal health services<br>Not visiting health facility<br>Could not get treatment for diseases<br>TBA is not attending delivery<br>Unable to buy medicines<br>Development partners not monitoring health facilities<br>Difficulty accessing care by disabled<br>Health facility closed<br>Health workers not available<br>Immunization stopped<br>Increase in self-medication	Unavailability of health services, immunization disruptions, and difficulties accessing maternal health services	Stakeholders highlight the impact on disabled individuals, while the literature focuses more on general access and utilization issues

	Unmet needs for family planning<br><br>Unmet needs for family planning among women in extended postpartum period<br><br>Unsure of availability of services<br><br>Ability of CHW to provide care<br><br>Access to health services<br><br>Access to medicine / medical treatment<br><br>Access to water, soap, and sanitizer in the health facilities<br><br>Availability of health professionals<br><br>Availability of health workers for childbirth<br><br>Availability of maternal health services<br><br>Elders access to physician<br><br>Health services utilization before COVID-19<br><br>Mass administration of medical campaigns to control Neglected Tropical Diseases<br><br>Providing immunization<br><br>Proximity to health facility	Lack of delivery kits for TBAs		

	Use of medicines<br><br>Utilization of Antenatal Care<br><br>Utilization of maternal health services during the COVID-19 restrictions			
**Poverty, hunger, and lack of food**	Difficulty in storing food as advised<br><br>Being sick / injured<br><br>Challenged by reduced economic support (cultural expectations)<br><br>Economic impact of the pandemic<br><br>Elders risk of poverty<br><br>Elders' children financial struggles<br><br>Food insecurity<br><br>Household skipped meals because of lack of food<br><br>Individual lack of money<br><br>Lack funds for transportation<br><br>Lack of electricity<br><br>Poverty	No financial support from relatives<br><br>Poverty<br><br>Shortage of food supply<br><br>Increased poverty<br><br>Lack of basic needs<br><br>Lack of hygiene<br><br>Less food to eat<br><br>Malnutrition<br><br>Men unable to provide family needs<br><br>More demand for food at home<br><br>More hunger	Shortage of food supply, increased poverty, and economic struggles	Stakeholders highlight the increased demand for food at home and men's difficulty to provide food for their households. The literature covers a range of factors like affordability and access to particular food products (staple foods, animal sourced food). The literature also emphasizes the financial constraints of elders and adolescents such as elders being dependent on intergenerational support in the absence of pensions.

	Retired elders' dependency on intergenerational support for financial security<br><br>Rural elders not being beneficiaries of pension scheme<br><br>Rural students' financial constraints<br><br>Shortage of food supply<br><br>Access to staple food<br><br>Adolescent food consumption<br><br>Affordability of food (ability to afford food for their families)<br><br>Affordability of preventive tools (masks, soap)<br><br>Food consumption<br><br>Have health insurance<br><br>Higher-income<br><br>Rural adolescent consumption of animal sourced food			
**Stress and mental health problems**	Community health worker (CHW) concern about their children's education<br><br>Elders feeling left out/excluded<br><br>Elders' loneliness / feeling isolated	Stress and worries<br><br>Feeling socially isolated<br><br>Increase in suicide	Increased psychological distress and feelings of isolation are common factors	Stakeholders mention challenges like domestic violence, increased burden on parents, and suicidal behaviour. The

	Female CHW bringing children to work – stress at work<br><br>Female CHW parental distress<br><br>Psychological distress<br><br>Women are anxious about their childbirth<br><br>Women loneliness in childbirth<br><br>CHW pride	Increased mental health problems<br><br>More burden on parents<br><br>More household responsibility on men<br><br>More household responsibility on women		literature focuses more on mental health related to community health workers and elders.
**Fear and misconceptions about COVID**	Belief that vaccine will not work<br><br>Concern for safety/ fear of infecting older people<br><br>Disbelief about the existence of COVID-19<br><br>Distrust in government, health organizations, and medical doctors<br><br>Early predictions about the pandemic in SSA<br><br>Elders fear of getting infected<br><br>Fear and stigma of others thinking you are COVID positive<br><br>Fear of contracting COVID-19 at the	Constant media report of COVID-19<br><br>People feeling insecure<br><br>Rumours on covid-19<br><br>Stigma against COVID patients<br><br>Fear of contracting COVID-19<br><br>Fear of death<br><br>Fear of each other<br><br>Lack of awareness on	Both mention the fear and stigma around getting COVID-19 and misconceptions about the virus and vaccines.	Stakeholder data emphasizes rumors, fear of death, and insecurity, while the literature focuses on government distrust and awareness campaigns. Literature emphasizes fear of infecting elders and older parents.

	health facility<br><br>Fear of contracting COVID-19 in transportation<br><br>Fear of getting infected<br><br>Fear of vaccine safety/ side effects<br><br>Fear of infecting older parents<br><br>Inadequate support from government to boost access to health information<br><br>Misinformation about COVID-19<br><br>Perceived risk of outbreak<br><br>Too many conflictive publications about COVID-19<br><br>Awareness campaigns<br><br>Awareness/ knowledge of COVID-19<br><br>Awareness/ knowledge of COVID-19 preventive measures<br><br>COVID-19 communication in native language (ex. Yoruba)<br><br>COVID-19 information source credibility<br><br>Information on COVID-19 through interpersonal communication<br><br>Ministry of health educational material	COVID-19<br><br>Misconception on covid vaccine			

	Perception of severity of COVID-19<br><br>Perceived susceptibility to COVID-19 infection<br><br>Relatable COVID communication/information<br><br>Understanding COVID-19 information/ communication			
**Lockdown**	More stringent and prolonged COVID-19 restriction policies<br><br>COVID-19 restrictions / mitigation strategies<br><br>Lockdown and movement restrictions<br><br>State borders closed after COVID<br><br>Movement restrictions<br><br>Lockdown<br><br>Lack of transport	Borders closed<br><br>No tourism<br><br>No transport<br><br>Restriction of movement<br><br>Filling station closed<br><br>LGA secretariat closed<br><br>Lockdown<br><br>Men couldn't come back home	Both include movement restrictions, border closures, and business closures.	Stakeholders provide details on elements of the lockdown such as the closure of public offices and the travel bans effect on tourism, while the literature focuses more on the broader lockdown policies and movement restrictions
**Reduced businesses and jobs**	Concern about income loss<br><br>Farming disruption	Businesses/trade stopped<br><br>No access to farm implements	Both highlight market closures and loss of jobs and income.	The stakeholder data includes more nuanced factors like loss of farm implements and government revenue,

	Highly interdependent/ interconnected rural-urban linkages<br><br>Homemaker occupation<br><br>Income loss<br><br>Job loss<br><br>Loss in income source<br><br>Markets/shops closed<br><br>Non-farm business closure<br><br>Economic activities<br><br>Husband employment<br><br>Market diversity<br><br>Market structure<br><br>Remittances	No buyers<br><br>No home/petty trade<br><br>No production of goods<br><br>Not going to farm<br><br>Poor harvest<br><br>Street hawking stopped<br><br>Decreased government revenue<br><br>Jobs lost<br><br>Markets/shops closed		while the literature includes details like loss of remittances and reduced market diversity.
**Reduced education**	Adolescents not getting any education<br><br>Difficulty catching up with education<br><br>Lack of e-learning platforms<br><br>Lack of parental support for rural students	No religious education<br><br>Poor performance by students<br><br>School/madrasah closed<br><br>Teachers absent	Both mention school closures, unavailable teachers, and the difficulties students face with remote e-learning.	Stakeholder data highlights religious education such as the increased burdens on traditional religious schools (madrasahs), while the literature includes the role of parents in children's

	Rural students’ lack of skills/digital literacy<br><br>Rural students’ preference for face-to-face learning<br><br>School closures<br><br>Unavailability of teachers to support students in the use of e-learning<br><br>Adolescent education from other channels<br><br>Adolescent self-assessed ability to learn<br><br>Access to digital learning among rural students<br><br>Parents teaching children alternative skills<br><br>Student awareness of digital learning intervention by government<br><br>Student learning via radio and television programmes	Two shifts in schools<br><br>Delay in education<br><br>Increase in school dropout<br><br>More burden on madrasah		education, digital literacy, digital learning platforms, and adolescents education from alternative channels.
